# Extracellular vesicle-associated tyrosine kinase-like orphan receptors ROR1 and ROR2 promote breast cancer progression

**DOI:** 10.1186/s12964-023-01186-1

**Published:** 2023-07-10

**Authors:** Barnabas Irmer, Janes Efing, Lea Elisabeth Reitnauer, Allegra Angenendt, Saskia Heinrichs, Antonia Schubert, Matthias Schulz, Claudia Binder, Joke Tio, Uwe Hansen, Christiane Geyer, Mirjam Gerwing, Annalen Bleckmann, Kerstin Menck

**Affiliations:** 1 University Hospital Münster, Dept. of Medicine A, Albert-Schweitzer-Campus 1 D3, 48149 Münster, Germany; 2 West German Cancer Center, University Hospital Münster, Münster, Germany; 3 Division Signaling and Functional Genomics, German Cancer Research Center (DKFZ) and Heidelberg University, Heidelberg, Germany; 4 Dept. of Medical Oncology, University Hospital Heidelberg, National Center for Tumor Diseases (NCT), Heidelberg, Germany; 5 Dept. of Hematology/Medical Oncology, University Medical Center Göttingen, Göttingen, Germany; 6 Dept. of Obstetrics and Gynaecology, University of Münster, Münster, Germany; 7 Institute for Musculoskeletal Medicine, University of Münster, Münster, Germany; 8 Clinic for Radiology, University of Münster, Münster, Germany

## Abstract

**Background:**

Extracellular vesicles (EVs) harbor a plethora of different biomolecules, which they can transport across cells. In cancer, tumor-derived EVs thereby support the creation of a favorable tumor microenvironment. So far, EV uptake and cargo delivery into target cells have been regarded as the main mechanisms for the pro-tumoral function of EVs. To test this hypothesis, we investigated the fate of the oncogenic transmembrane Wnt tyrosine kinase-like orphan receptor 1 and 2 (ROR1, ROR2) delivered via distinct EV subpopulations to breast cancer cells and aimed to unravel their impact on tumor progression.

**Methods:**

EVs were isolated by differential ultracentrifugation from cell culture supernatant as well as plasma samples from healthy individuals (*n* = 27) and breast cancer patients (*n* = 41). EVs were thoroughly characterized by electron microscopy, nanoparticle tracking analysis, immunoblot, and flow cytometry. ROR transfer to target cells was observed using microscopy-based assays and biodistribution experiments were conducted in syngeneic mice. EV impact on cancer cell migration and invasion was tested in functional assays.

**Results:**

We observed that the supernatant of ROR-overexpressing cells was sufficient for transferring the receptors to ROR-negative cells. Analyzing the secretome of the ROR-overexpressing cells, we detected a high enrichment of ROR1/2 on large and small EVs, but not on large oncosomes. Interestingly, the majority of ROR-positive EVs remained attached to the target cell surface after 24 h of stimulation and was quickly removed by treatment with trypsin. Nonetheless, ROR-positive EVs increased migration and invasion of breast cancer cells, even after chemically inhibiting EV uptake, in dependence of RhoA downstream signaling. In vivo, ROR-depleted EVs tended to distribute less into organs prone for the formation of breast cancer metastases. ROR-positive EVs were also significantly elevated in the plasma of breast cancer patients and allowed to separate them from healthy controls.

**Conclusions:**

The oncogenic Wnt receptors ROR1/2 are transferred via EVs to the surface of ROR-negative cancer cells, in which they induce an aggressive phenotype supporting tumor progression.

Video Abstract

**Supplementary Information:**

The online version contains supplementary material available at 10.1186/s12964-023-01186-1.

## Background

Breast cancer is the most common cancer among women worldwide with more than 2.2 million new cases diagnosed in 2020 [1]. As true for most solid tumor entities, metastasis development in the course of the disease usually limits survival due to the lack of therapeutic options. One of the molecular pathways linked to breast cancer progression and metastasis is the Wnt signaling pathway [2]. In mammals, 19 distinct secreted Wnt ligands may bind a subset of ten Frizzled (Fzd) receptors that interact with multiple co-receptors including the receptor tyrosine kinase-like orphan receptor 1 and 2 (ROR1 and ROR2) [2]. While both ROR1 and ROR2 are only detectable at low levels in healthy tissue, they are found overexpressed in breast cancer primaries [3] as well as metastases [4]. High expression was linked to increased cancer cell invasiveness and poor patient survival [5, 6], highlighting their tumor-promoting role in this entity. The intracellular downstream signaling initiated upon Wnt/ROR binding is multi-faceted and can involve an initial recruitment of a DVL/RHO/DAAM1 complex that mediates the subsequent activation of the RHO/ROCK pathway [3]. ROR1/2 downstream signaling can thus lead to cytoskeletal rearrangements affecting cell motility and morphology.

Although Wnts had traditionally been described as secreted proteins, they were shown to associate with small extracellular lipid nanoparticles secreted by all living cells, the so-called extracellular vesicles (EVs) [7, 8]. The association of the hydrophobic Wnt proteins to EVs is thought to increase their stability in the extracellular milieu and thus enhance their long-distance signaling capacity. EVs are mediators of intercellular communication across different body cells. They can be sub-categorized into three main classes based on their size: I) small EVs (sEVs) with a diameter of 50 – 150 nm, II) large EVs (lEVs) with a diameter of 100 – 1000 nm, and III) large oncosomes (LOs) with a diameter of 1000 – 10,000 nm that are exclusively shed from the surface of cancer cells. In addition, dying cells release apoptotic bodies with a diameter of 500 – 4000 nm [9]. Tumor cell-derived EVs have emerged as critical messengers in cancer progression. They are associated with several key features of the disease such as angiogenesis, immunosuppression, local tumor growth, as well as cancer cell dissemination and metastasis [10]. Since EVs are highly abundant in almost all human body fluids including blood [9], they may also act on cells at distant tissues to contribute to pre-metastatic niche formation and thus support tumor cell spreading [11, 12]. Their pro-tumoral function is linked to their cargo which comprises a plethora of pro-tumorigenic nucleic acids, lipids, and proteins (e.g. Wnt proteins, KRAS, EGFR, MMPs, EpCAM, HSP family members) which they can shuttle to surrounding tumor as well as stroma cells to foster transformation and aggressive phenotypes in recipient cells [13]. Two distinct mechanisms for EV protein interactions with target cells have been described: 1) Binding of cognate receptors and ligands on the surface of EVs and target cells that can initiate intracellular signaling cascades [8, 14, 15], and 2) horizontal transfer of EV proteins by either membrane fusion or endocytic internalization of EVs [16–18]. Internalization and transfer of biologically active proteins are still believed to be the main route [19], and the dependence of vesicle uptake for EV function is rarely investigated.

In the present study, we used breast cancer cell lines as well as patient samples to investigate whether the two Wnt co-receptors ROR1 and ROR2 can be spread via EVs. We aimed to elucidate the fate of the receptors at the recipient cells and analyze whether EV-incorporated RORs retain their tumor-promoting properties to induce an aggressive phenotype in surrounding breast cancer cells.

## Methods

### Cell lines and conditioned medium

Human MCF-7 and MDA-MB-231 (both DSMZ) and mouse 4T1 (ATCC) breast cancer cells were cultured in RPMI-1640 supplemented with 10% heat-inactivated (56 °C, 30 min) fetal calf serum (FCS, Anprotec). All cultured cells were routinely tested to exclude contamination with Mycoplasma. To generate MCF-7 cells with overexpression of ROR1 or ROR2, cells were transfected using the Fugene HD transfection system (Invitrogen). Stable clones were selected by geneticin, zeocin or hygromycin selection (750 µg/ml, 100 µg/ml or 300 µg/ml). 4T1 and MDA-MB-231 CRISPR control or ROR1 knockout (KO) cells were generated by transiently transfecting cells with the PX461-GFP and -mCh plasmid encoding for the Cas9n (D10A nickase mutant) with/without sgRNAs targeting *ROR1* exon 1 (sequences for human *ROR1*: 5’-CGGGACGCGCCCGCCGCTCC-3’ and 5’-GCGCTGCTGCTGGCCGCACG-3’; sequences for mouse *Ror1*: 5’-CCGCGGGACGCGCCCGCCAC-3’ and 5’-GCTGCTGGCCGCGCTGCTGC-3’). Double-positive cells were sorted by flow cytometry and grown as single-cell clones. Conditioned medium was generated by washing the cells twice in PBS followed by incubation in RPMI + 10% EV-depleted FCS overnight. The next day, the supernatant was harvested and either briefly centrifuged for 5 min at 500 × g to remove residual cells or centrifuged at 143.000 × g to deplete all EVs.

### Vectors

The pcDNA3.1/Zeo( +) (pcDNA) empty vector was obtained from Invitrogen and the pROR1 vector from Sinobiological (HG13968-UT). The pCMV empty vector was generated by KpnI and XbaI digestion from plasmid HG15755-UT (Sinobiological). The pROR2 vector was kindly provided by Alexandra Schambony. The pSpCas9n(BB)-2A-GFP (PX461-GFP) vector was a gift from Feng Zhang (Addgene plasmid #48,140; http://n2t.net/addgene:48140; RRID:Addgene_48140) [20] and pSpCas9n(BB)-2A-mCh (PX461-mCh) was kindly provided by Pascale Zimmermann.

### Patients

EDTA-anticoagulated blood was collected from patients with confirmed breast cancer (*n* = 41) and healthy individuals (*n* = 27). Patient and control characteristics are summarized in Tables 1 and 2. Samples for EV isolation were obtained prior to treatment to minimize possible contamination with apoptotic bodies. Due to the limited amount of lEVs in some samples, it was not possible to determine the expression of all tumor proteins in all samples by flow cytometry.

**Table 1:** Characteristics of the study cohort

Subgroup	*n* =	Age median in years [95% CI]	Male sex *n* = [frequency]
Breast cancer (BC)	41	57 [52–61]	1 [0.024]
Healthy (CTL)	27	35 [31-40]	3 [0.111]

**Table 2:** Patient characteristics

**Breast cancer patients**		***n* = **
Stage	I	0
	II	7
	III	5
	IV	26
	unknown	3
Molecular subtype	Luminal A-like	9
	Luminal B-like Her2-neg	13
	Luminal B-like Her2-pos	7
	Her2-enriched	7
	basal-like	5
Histological subtype	No specific type (NST)	31
	invasive lobular carcinoma	5
	invasive papillary carcinoma	1
	mixed	3
	unknown	1

### Isolation and staining of EVs

We have submitted all relevant data of our EV isolation and characterization experiments to the EV-TRACK knowledgebase (EV-TRACK ID: EV230059) [21]. For EV isolation, cancer cells were washed twice with PBS and cultured for 24 h in RPMI-1640 supplemented with EV-depleted FCS (ultracentrifuged for 16 h at 153,700 × g and filtered through a 0.2 µm filter). Supernatants were collected and centrifuged at 500 × g for 5 min to remove residual cells and debris. Subsequently, LOs were pelleted by centrifugation at 1,500 × g for 15 min, lEVs at 17,000 × g for 30 min, and sEVs at 143,000 × g for 90 min. Isolation of EVs from patient samples was performed as described previously [22]. Briefly, 10–15 ml peripheral blood were collected in tubes containing EDTA (1.6 mg/ml blood, Sarstedt) and were processed within 30 min of blood withdrawal. To obtain plasma samples, the blood was centrifuged for 15 min at 1,200 g and passed through a valve filter (Seraplas, Sarstedt). Plasma was centrifuged for 15 min at 1,500 g to remove residual platelets and was stored at − 20 °C. lEVs were pelleted at 17,000 × g for 30 min and the supernatant was filtered through a 0.2 µm filter prior to ultracentrifugation at 143,000 × g for 90 min to pellet sEVs. All EV pellets were washed once in PBS and stored in PBS for subsequent experiments. For DiR staining, EVs were incubated in 2 µM DiR (1,1-dioctadecyl-3,3,3,3-tetramethylindotricarbocyanine iodide, #D12731, Thermo Scientific) for 15 min at RT, pelleted as described above and resuspended in PBS for subsequent experiments. Dye-only samples were processed accordingly but without the addition of EVs.

### Animal experiments

Female BALB/c mice (age 8–12 weeks, Charles River) were kept in groups of 5 at an ambient temperature of 23—26 °C and relative humidity of 45 – 65% under a 12 h light–dark-cycle with ad libitum access to food and water and alternating play and embedding material. Mice were dehaired using a razor and depilatory cream on the back and bottom the day before the injection to avoid artifacts in the fluorescence reflectance imaging (FRI) scan. Animals were injected with 100 µl PBS containing 100 µg of DiR-labeled EVs isolated from 4T1 wildtype (WT) or ROR1 KO cells and fluorescence signals of the organs were recorded by FRI scans as described previously [23] after sacrificing the mice 24 h post injection.

### Nanoparticle tracking analysis (NTA)

EV size distribution and concentration were analyzed with the ZetaView PMX120-S device (ParticleMetrix) equipped with a 640 nm laser and a CMOS camera. Samples were diluted in PBS to obtain a concentration of 50 – 400 particles per frame. Particles were tracked in short videos of 1 s at eleven distinct positions and particle concentration and size were calculated with the ZetaView software (version 8.02.31).

### Transmission electron microscopy

Pellets containing isolated EVs were fixed in 2% (v/v) formaldehyde and 2.5% (v/v) glutaraldehyde in 100 mM cacodylate buffer, pH 7.4, at 4 °C overnight. After washing in PBS, samples were post­fixed in 0.5% (v/v) osmium tetroxide and 1% (w/v) potassium hexacyanofer­rate (III) in 0.1 M cacodylate buffer for 2 h at 4 °C followed by washing with distilled water. After dehydration in an ascending ethanol series from 30 to 100% ethanol, specimens were incubated twice in propylene oxide for 15 min each and embedded in Epon using Eppendorf safe-lock tubes. Ultrathin sections were cut with an ultramicrotome, collected on copper grids, and negatively stained with 2% uranyl acetate for 10 min. Electron micrographs were taken at 60 kV with a Phillips EM-410 electron microscope using imaging plates (Ditabis).

### Western blot

Cells were lyzed in RIPA buffer (50 mM Tris, 150 mM NaCl, 0.1% SDS, 0.5% sodium deoxycholate, 1% Triton X-100, pH 7.2) supplemented with protease (Sigma) and phosphatase (Roche) inhibitors. Up to 50 µg of cell lysate or EVs were subjected to SDS-PAGE (8–12% gels) and subsequently blotted onto nitrocellulose. Membranes were blocked for 1 h at RT in 3% bovine serum albumin in TBS-T (137 mM NaCl, 20 mM Tris pH 7.6, 0.1% (v/v) Tween-20) and incubated with specific primary antibodies overnight at 4 °C. Antibodies were directed against ROR1 (#16540, 1:1000), GM130 (#12480, 1:1000), HDAC1 (#2062, 1:1000, all three from cell signaling), ROR2 (#sc-98486, 1:1000), GAPDH (#sc-32233, 1:1000), α-Actinin-4 (#sc-390205, 1:1000), RGAP1 (#sc-271110, 1:1000), CK18 (#sc-6259, 1:1000), TSG101 (#sc-7964, 1:1000), CD81 (#sc-166028, 1:1000), Rock1 (#sc-17794, 1:500), Rock2 (#sc-398519, 1:500), RhoA (#sc-418, 1:1000), ApoA1 (#sc-376818, 1:1000), ApoB, (#sc-393636, 1:1000), albumin (#sc-271605, 1:1000), ꞵ-actin (#sc-47778, 1:2000, all from santa cruz biotechnology), Alix (#12422–1-AP, 1:1000, Proteintech), or Syntenin-1 (#ab133267, 1:2000, abcam). Membranes were incubated with respective HRP-labeled secondary antibodies (#7074, #7076, 1:10,000, cell signaling) for 1 h at RT and chemiluminescence was detected using West Pico (Thermo Scientific) or Clarity Max (Bio-Rad) ECL substrate at the ChemoStar Touch Imager (Intas). ImageJ software (version 1.52p) was used for densitometric quantification.

### Immunofluorescence and co-localization experiments

Cells were seeded onto glass coverslips and allowed to adhere overnight. After EV stimulation (10 µg/ml) for 4 or 24 h, cells were washed twice with PBS and fixed in 4% PFA for 15 min. Unspecific binding sites were blocked in PBS + 0.3% BSA + 0.05% saponin and cells incubated with primary antibodies directed against ROR1 (#357803, 1:100, Biolegend), ROR2 (#FAB20641G, 1:100, R&D systems), EEA1 (#3288, 1:100, cell signaling), EpCAM (#ab71916, 1:100, abcam) or LAMP2 (#PA1-655, 1:100, Invitrogen) for up to 4 h at RT. Cells were incubated with anti-rabbit secondary antibodies conjugated to Alexa Fluor 488 or Alexa Fluor 594 (#406416 or #406418, 1:2000, Biolegend) and mounted in Fluoroshield mounting medium with DAPI (Abcam). Coverslips were imaged on the LSM 800 AiryScan confocal laser scanning microscope (Zeiss). To assess the extent of ROR-EVs extracellularly adhered to target cells, MCF-7 cells were stimulated with EVs (10 µg/ml) for 24 h. Cells were washed twice with PBS (= control) or treated with 0.01% trypsin/EDTA for 90 s at 37 °C followed by one washing step with 5% FCS in PBS. Cells were washed once in PBS and immunostained for ROR2 as described above. For EV uptake studies, adherent MCF-7 cells seeded on glass cover slips were pretreated with Dynasore (12.5 µM) for 2 h prior to the addition of PKH26-labeled EVs (4 µg/ml) for 24 h. After two PBS washing steps and fixation with 4% PFA, cells were mounted in Fluoroshield mounting medium with DAPI (Abcam). Cellular EV infiltration was imaged on the LSM 800 AiryScan confocal laser scanning microscope (Zeiss) and quantified by calculating the ratio between PKH26 signal intensity and cell number per image. PKH26 signal intensities were measured using the Image J software (version 1.52p).

### Cell invasion

Cancer cell invasion was analyzed by a modified Boyden chamber assay [24]. Briefly, MCF-7 cells were seeded in triplicates onto a polycarbonate membrane (10 µm pore diameter, Pieper Filter) coated with basement membrane extract (R&D systems) in the upper wells of the chamber and were stimulated with EVs (1 µg/ml) for 96 h. The number of invasive cells in the lower wells of the chamber was counted and related to an unstimulated control. For inhibition of EV uptake, cells were pre-incubated with Dynasore (12.5 µM) for 2 h prior to EV addition, and cell invasion was quantified after 48 h due to the toxicity of the inhibitor upon longer incubation times.

### Cell migration

MCF-7 cells were seeded into the wells of a 24-well plate (2 × 10^4^ cells/well) coated with basement membrane extract (R&D systems) and allowed to adhere overnight. Cells were stimulated with EVs (10 µg/ml) for 8 h and every 10 min one picture was captured at two distinct positions per well using the BZ-X800 fluorescence microscope (Keyence). Single cells (10 – 20 per condition) were tracked using the Image J software (version 1.52p).

### Flow cytometry

Up to 2 µg of lEVs were blocked for 15 min at RT in 20 µl PBS + 1% EV-depleted FCS and stained with fluorescently-labeled antibodies directed against EpCAM (#324208, 30 ng/sample), ROR1 (#357803, 2.5 ng/sample, both from Biolegend), ROR2 (#FAB20641G, 12.5 ng/sample, R&D systems), or corresponding isotype controls at the same concentration (#400321 and #400113 from Biolegend, #IC003G from R&D systems) for 20 min at RT. Fluorescence was recorded on the FACSymphony A1 (BD) flow cytometer and the percentage of positive events in the lEV gate was determined in relation to the respective isotype control. The submicron bead calibration kit (#832, Bangs Laboratories) was used to define the gate for lEVs. For EV uptake studies, cells were pretreated with Dynasore (12.5 µM) for 2 h prior to the addition of DiR-labeled EVs (10 µg/ml) for 24 h. Cells were washed twice with PBS and the mean fluorescence DiR intensity of single cells was recorded on the FACSymphony A1 (BD) flow cytometer. Data was analyzed with FACS Diva (version 9.0.2, BD) and FlowJo (version 10.6.1, BD) software. Flow cytometer acquisition settings were maintained for all samples, including triggering threshold, voltages, and flow rate.

### Statistical analysis

All experiments were carried out in at least three biologically independent replicates. Statistical analyses were performed with GraphPad Prism (v8.4.2) or SPSS (IBM, v29.0) using a two-sided student’s t-test for comparison of two groups or one-way ANOVA for comparison of multiple groups unless indicated otherwise. Single outliers were identified using the Grubbs’ test with a significance level of 5%. Correlation analyses were performed with Spearman's rank correlation. For evaluation of the diagnostic potential of the lEV tumor proteins, receiver operating characteristic (ROC) curves were generated, and the area under the curve (AUC) with 95% confidence interval (CI) was assessed by the Wilson/Brown method. The cut-off-values were determined based on the median of the antigen expression on IEVs in breast cancer patients. *P*-values < 0.05 were considered significant and are labeled as *p < 0.05, **p < 0.01, ***p < 0.001 and ****p < 0.0001. All figures in the current study were generated with GraphPad Prism (v8.4.2) or OMERO (v5.14.1) in case of the immunofluorescence images.

## Results

### Conditioned medium from ROR1/2-overexpressing cells induces ROR1/2 uptake in breast cancer cells

**Fig. 1:**
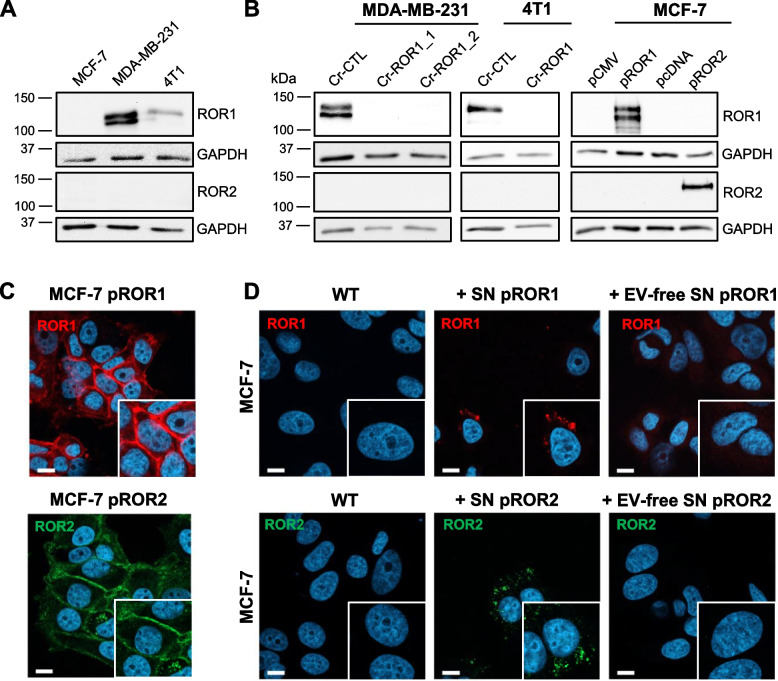
Supernatant of ROR1/2-overexpressing MCF-7 cells is sufficient to transfer receptors to originally ROR-negative cells. **A** Western blot: Expression of ROR1 and ROR2 in breast cancer cells. **B** Western blot: Expression of ROR1 and ROR2 in MDA-MB-231 and 4T1 CRISPR control (Cr-CTL) or ROR1 KO (Cr-ROR1) cells or MCF-7 cells stably overexpressing either an empty vector (pCMV, pcDNA) or ROR1/ROR2 overexpression construct (pROR1/pROR2). **C** Immunofluorescence: Localization of ROR1 or ROR2 in MCF-7 pROR1 or pROR2, respectively, was imaged by confocal microscopy. **D** ROR-negative MCF-7 wildtype (WT) cells were stimulated with supernatant (SN) of pROR1 or pROR2 cells and ROR1/2 signals were visualized by immunofluorescence and confocal microscopy. To generate EV-free SN, the SN was centrifuged for 2 h at 143,000 × g. Scale bar: 10 µm

To study the extracellular spreading of ROR1 and ROR2 in breast cancer, we chose human MCF-7 and MDA-MB-231 as well as murine 4T1 breast cancer cells as model cell lines. While MCF-7 cells did not express any of the two receptors, both MDA-MB-231 and 4T1 carried detectable levels of endogenous ROR1, but were negative for ROR2 (Fig. 1A). Using CRISPR/Cas9-mediated knockout (KO), we generated MDA-MB-231 and 4T1 cells with complete ROR1 KO (Fig. 1B). Although it has been shown that both receptors can compensate for each other [25], ROR1 KO did not lead to a compensatory upregulation of ROR2 in neither of the two cell lines (Fig. 1B). MCF-7 cells, in contrast, were stably transfected with an overexpression vector for ROR1 (pROR1) or ROR2 (pROR2), which induced expression of either receptor predominantly at the cell membrane of the transfected cells (Fig. 1C) comparable to the expression pattern of endogenous ROR1/2 in MDA-MB-231 cells (Suppl. Figure 1A). To test whether ROR1/2 can be secreted into the supernatant and spread to surrounding cells, we stimulated ROR-negative MCF-7 wildtype cells for 24 h with conditioned medium isolated from MCF-7 pROR1 and pROR2 cells and used immunofluorescence to detect ROR signals in stimulated cells. Indeed, confocal microscopy revealed signals of both ROR1 and ROR2 in recipient cells (Fig. 1D). Conditioned medium contains the whole secretome of the original cells, including soluble factors as well as EVs. To determine which of the two compartments was responsible for the observed ROR transfer, we depleted the conditioned medium from EVs by ultracentrifugation at 143,000 × g for 90 min prior to stimulation. Although the EV-depleted conditioned medium still contained all soluble proteins, ROR signals were no longer visible in stimulated MCF-7 cells (Fig. 1D). Similarly, conditioned medium from MDA-MB-231 cells transferred ROR1 expression to MCF-7 cells in an EV-dependent manner (Suppl. Figure 1B), albeit with weaker signals due to the comparatively low endogenous ROR1 levels in MDA-MB-231 cells. Taken together, these observations pointed to EVs as carriers of secreted ROR1/2.

### ROR1/2 are enriched on lEVs and sEVs

**Fig. 2:**
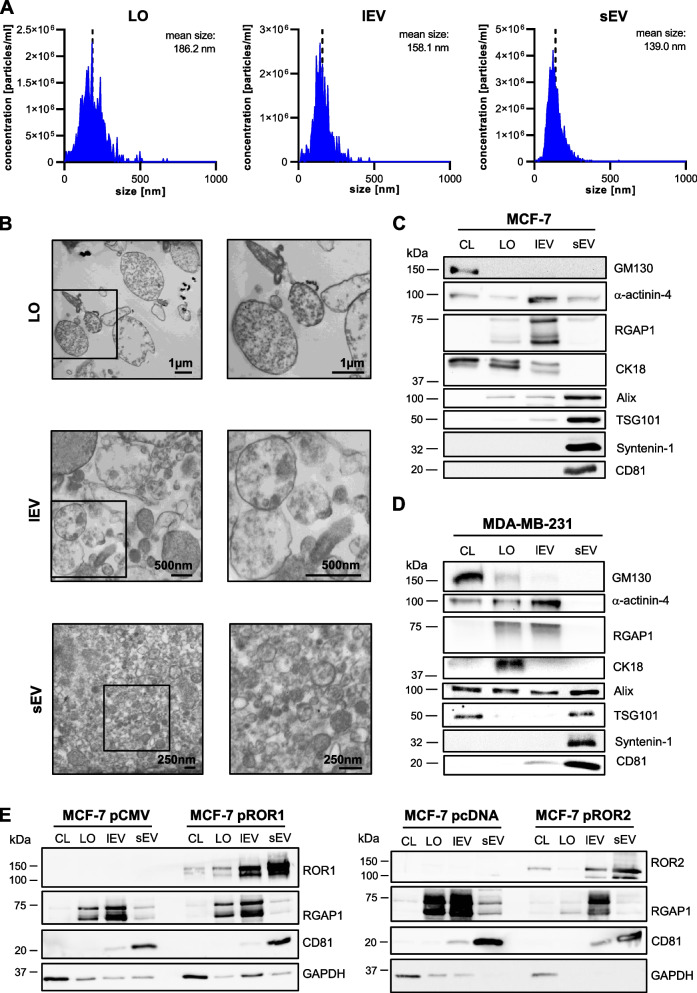
ROR1 and ROR2 are enriched on tumor-derived lEVs and sEVs. ***A*** NTA of the three different EV subpopulations harvested from MCF-7 wildtype cells. **B** TEM of the MCF-7 EV subpopulations. The image on the left displays a wide-field overview of the sample. The panel on the right contains a close-up of the area marked with a black box in the wide-field image. **C** + **D** Western blot: Characterization of EVs isolated from (**C**) MCF-7 and (**D**) MDA-MB-231 wildtype cells for common EV markers. GM130 is shown as a negative marker. Equal amounts of protein were loaded in every lane. **E** Western blot: ROR1 or ROR2 expression on EVs harvested from MCF-7 cells transfected with ROR1/2-overexpression vectors (pROR1/pROR2) or respective empty vectors (pCMV/pcDNA). CD81 served as sEV marker, RGAP1 as marker for LOs and lEVs. Equal amounts of protein were loaded in every lane

In order to confirm that EVs can indeed transport ROR1 and ROR2, we isolated the main EV subpopulations, LOs, lEVs, and sEVs, from the conditioned medium of the three breast cancer cell lines by using differential ultracentrifugation. NTA of MCF-7 and 4T1 EVs showed the successful enrichment of three distinct EV subpopulations that differed in size (Fig. 2A, Suppl. Figure 2A). Further characterization of the EVs by TEM confirmed the difference in size and revealed that, in particular, lEVs comprised a very heterogeneous population of EVs with sizes ranging from around 150 nm up to 1 µm. At the same time, LO preparations seemed to contain a significant portion of cell debris next to vesicular structures (Fig. 2B, Suppl. Figure 2B). The sEV samples were composed of relatively homogeneous vesicles of smaller size compared to lEVs or LOs (Fig. 2B, Suppl. Figure 2B). Proteomic analysis of EVs from MCF-7, MDA-MB-231 and 4T1 cells showed that sEVs highly expressed the typical markers Syntenin-1, Alix, TSG101, and CD81 (Fig. 2C + D, Suppl. Figure 2C). In contrast, lEVs were enriched in the markers α-actinin-4 and RGAP1 [26] and LOs in CK18 [27] which were all detectable in only minor amounts on sEVs. The Golgi protein GM130 or the histone deacetylase HDAC1 were absent on MCF-7 or 4T1 EVs, respectively, arguing against significant contamination of EV preparations with intracellular vesicles. Likewise, EV populations from MCF-7 and MDA-MB-231 cells were detected negative concerning lipoprotein impurities as shown for ApoA1, ApoB and albumin expression (Suppl. Figure 3). A slight expression of GM130 was detected on LOs from MDA-MB-231 cells (Fig. 2D) which could underline the presence of cell debris in this specific EV subpopulation. Taken together, these results confirmed the successful isolation of three distinct EV subpopulations differing in size and enriched in specific cargo proteins.

First, analyzing the expression of endogenous ROR1 on EVs from MDA-MB-231 cells, we found a strong enrichment of the protein on sEVs, and, to a lesser extent, also on lEVs, compared to the cell lysate (Suppl. Figure 2D). ROR1 was not present on LOs from MDA-MB-231 cells. The same expression pattern was observed on EVs from 4T1 cells for ROR1 (Suppl. Figure 2E) and EVs from MCF-7 pROR1 and pROR2 cells for ROR1 as well as ROR2 (Fig. 2E). LOs from MCF-7 pROR1 carried minor amounts of ROR1, which were neglectable compared to ROR1 expression on lEVs and sEVs. Therefore, we concluded that high levels of both ROR1 and ROR2 can be exported to lEVs and sEVs, but not to LOs.

### ROR1/2 are transported on lEVs and sEVs to breast cancer cells and remain extracellularly attached to the target cell membrane

**Fig. 3:**
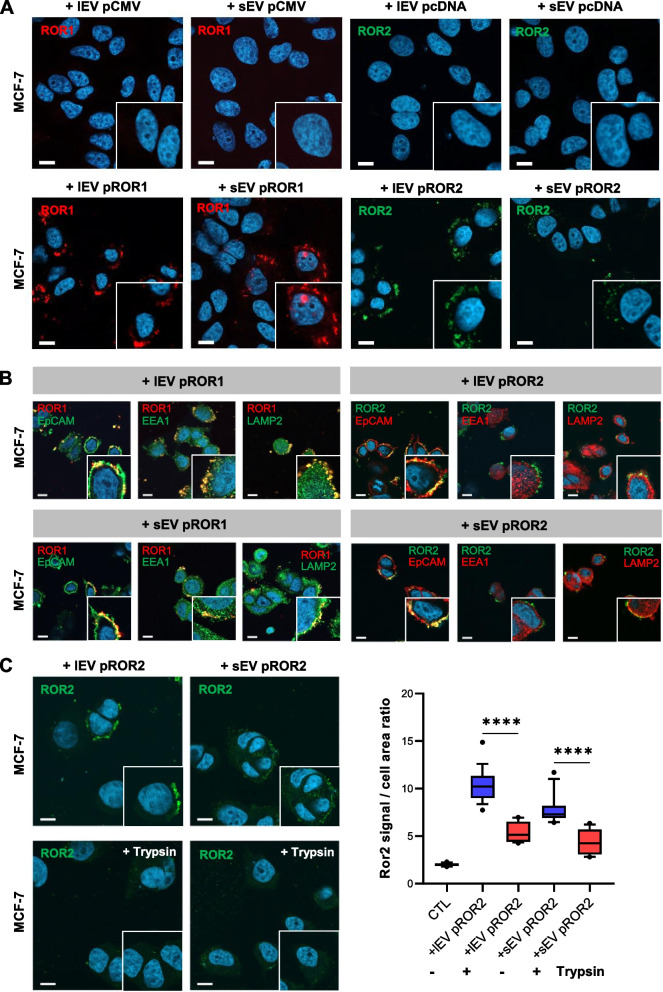
EV-delivered ROR1/2 mainly accumulates at the extracellular site of the target cell plasma membrane after 24 h. **A** Immunofluorescence: MCF-7 cells were stimulated for 24 h with 10 µg/ml lEVs or sEVs from empty vector control (pCMV/pcDNA) or ROR1/2 overexpressing (pROR1/pROR2) cells and ROR signals were visualized by confocal microscopy. Scale bar: 10 µm. **B** Confocal microscopy: Immunofluorescence-based co-localization of ROR1/2 with EpCAM, EEA1 or LAMP2 in MCF-7 cells stimulated for 24 h with lEVs (upper panel) or sEVs (lower panel) isolated from MCF-7 pROR1 or pROR2 cells. Scale bar: 10 µm. **C** MCF-7 cells were stimulated with EVs from pROR2 cells for 24 h, then treated with/without trypsin for 90 s at 37 °C (*n* = 3). Boxplots depict the median (line), the 25–75 percentiles (box) and the 10–90 percentiles (whiskers) of *n* = 15 quantified fields. Scale bar: 10 µm

Having detected high levels of vesicular ROR1 and ROR2 expression in breast cancer cells, we asked whether RORs can be transported to surrounding cells via EVs. To address this question, MCF-7 wildtype cells were stimulated for 24 h with 10 µg/ml lEVs and sEVs from MCF-7 pROR1/pROR2 cells or MDA-MB-231 Cr-ROR1 cells, respectively, and ROR signals were visualized by immunofluorescence. Using confocal microscopy, we detected ROR1 or ROR2 in initially ROR-negative MCF-7 cells upon stimulation with EVs from pROR1 or pROR2 cells but not with EVs isolated from the respective empty vector cells (Fig. 3A). Accordingly, EVs from ROR1-depleted MDA-MB-231 cells failed to transfer ROR1 expression contrary to control cell-derived EVs (Suppl. Figure 4A). Since ROR signals were mainly localized to the cell periphery, we performed co-immunostainings with markers for the different cellular compartments, in order to shed light on the intracellular localization of the EV-transported receptors in target cells. Because stimulations with MDA-MB-231 EVs transfered only low amounts of ROR1 to recipient cells, we limited these co-localization studies to pROR1 and pROR2 EVs. The transmembrane epithelial cell adhesion molecule (EpCAM) was chosen as a marker for the cell membrane, the early endosome antigen 1 (EEA1) for early endosomes and lysosome-associated membrane protein 2 (LAMP2) for late endosomal compartments. Interestingly, a major part of EV-delivered ROR2 co-localized with EpCAM after 4 h as well as 24 h of stimulation, while there was no co-localization with EEA1 or LAMP2 (Fig. 3B, Suppl. Figure 4B). For EV-trafficked ROR1, there was a clear co-localization with EpCAM, but to a lesser part, also with EEA1 and LAMP2 located at the cell periphery (Fig. 3B, Suppl. Figure 4B). These observations suggested that after EV stimulation for up to 24 h, a major part of the ROR-EVs might not be taken up into the target cells but localized to the cell membrane, in particular in the case of ROR2. In order to clarify whether ROR2-EVs are taken up or remain extracellularly attached to the recipient cell surface, we treated ROR2-EV-stimulated MCF-7 cells for 90 s with trypsin and detected ROR2 expression in target cells by using an antibody directed against the C-terminal part of ROR2 to exclude cleavage of the extracellular antibody recognition site by trypsin. The microscopic pictures revealed that trypsin treatment reduced EV-mediated ROR2 signals in recipient cells by around 60% (Fig. 3C), thus confirming that a major part of the ROR2-EVs remained attached to the extracellular part of the cell membrane and was not taken up for horizontal cargo delivery after 24 h.

### ROR-EVs induce cancer cell invasion via RhoA

**Fig. 4:**
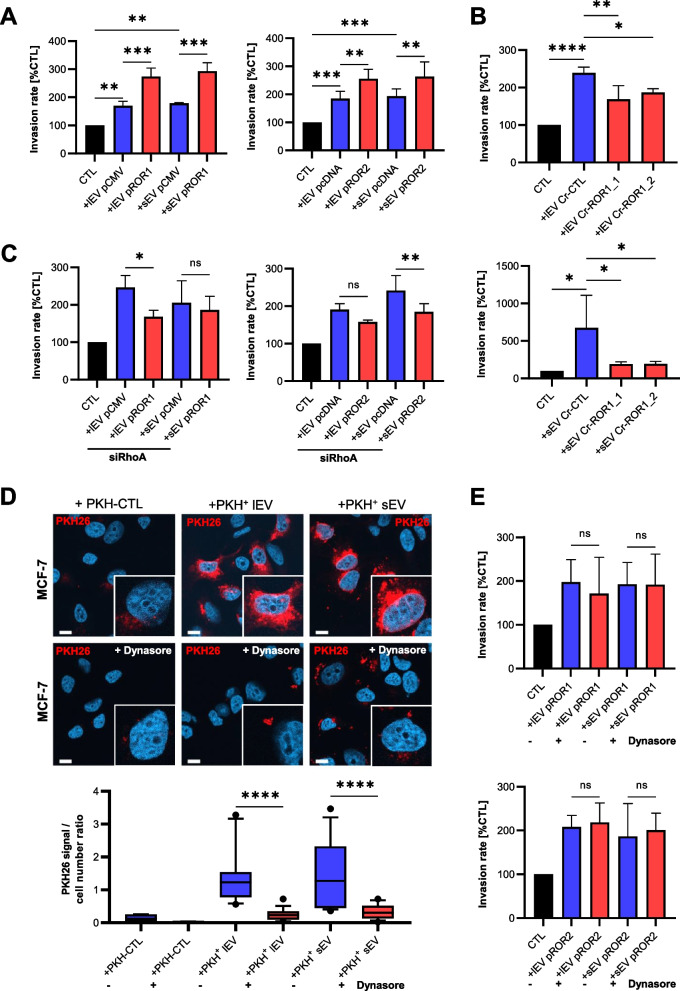
Stimulation with EVs derived from ROR1/2-overexpressing cells increases tumor invasion dependent on RhoA. **A + B** Boyden chamber invasion assays of MCF-7 cells after stimulation with (*A*) EVs isolated from MCF-7 empty vector (pCMV/pcDNA) or ROR1/2-overexpressing (pROR1/pROR2) cells or (*B*) EVs from MDA-MB-231 CRISPR control (Cr-CTL) or ROR1 KO (Cr-ROR1) cells (mean ± SD, *n* = 3). **C** Boyden chamber invasion assays of MCF-7 cells transfected with siRNA against RhoA (10 nM) 24 h prior to stimulation with EVs isolated from MCF-7 empty vector (pCMV/pcDNA) or ROR1/2-overexpressing (pROR1/pROR2) cells (mean ± SD, *n* = 2). **D** Immunofluorescence: MCF-7 cells were pre-treated with/without dynasore (12.5 µM) for 2 h and uptake of PKH26-labeled EVs or dye-only (PKH-CTL) into the cells was visualized by confocal microscopy (*n* = 3). Signals were quantified with ImageJ. Boxplots depict the median (line), the 25–75 percentiles (box) and the 10–90 percentiles (whiskers) of *n* = 15 quantified fields. Scale bar: 10 µm. **E** Boyden chamber invasion assay of MCF-7 stimulated with pROR1/pROR2 EVs after pre-treatment of the cells with/without dynasore (12.5 µM) for 2 h (mean ± SD, *n* = 3)

Next, we investigated whether, despite the only minor ROR-EV uptake, the delivered receptors can still induce functional Wnt/ROR signaling. Indeed, stimulation of MCF-7 cells with ROR-EVs from pROR1 as well as pROR2 cells led to a significant increase of cancer cell invasiveness in Boyden chamber assays (Fig. 4A). EVs from empty vector cells supported tumor invasion as well, but to a lesser extent compared to ROR-EVs, indicating that next to the RORs also other factors are involved in the invasion-promoting effect of tumor EVs. In line with these results, both lEVs as well as sEVs from MDA-MB-231 CRISPR control cells enhanced the invasiveness of MCF-7 cells, while this effect was significantly reduced for MDA-MB-231 ROR1 KO EVs (Fig. 4B). Analysis of single-cell migration of MCF-7 cells on extracellular matrix indicated that the invasion effect observed in Boyden chambers was linked to an enhanced migratory potential of the breast cancer cells, including an increase in migration speed and distance, upon stimulation with ROR-EVs (Suppl. Figure 5). In breast cancer cells, cellular expression of ROR1 and ROR2 has been linked to tumor invasion via Rho/Rock signaling [6, 28]. In line, in targets cells that have been exposed to siRNA-mediated knockdown of RhoA EV-incorporated ROR1 and ROR2 were shown to lose their augmented pro-invasive potential compared to ROR-negative control (Fig. 4C). Of note, upon RhoA depletion EVs from ROR1 and ROR2 overexpressing cells tended to induce target cell invasion even less compared to their respective empty vector control EVs. The pro-invasive effect of ROR-EVs did not seem to be mediated by increased RhoA levels which remained unchanged upon incubation of tumor cells with the EVs (Suppl. Figure 6A). However, stimulation of MCF-7 cells with lEVs from pROR1 cells slightly increased cellular levels of the RhoA downstream target Rock1 (Suppl Fig. 6A). This effect was neither observed with corresponding sEVs nor with EVs from pROR2 cells. In contrast, lEVs and sEVs from pROR2 cells seemed to slightly increase Rock2 expression in target cells, although this trend did not reach statistical significance (Suppl. Figure 6A). Likewise, in MCF-7 cells treated with MDA-MB-231-derived control and ROR1 KO EVs, we did not observe significant changes in total RhoA or Rock1/2 levels (Suppl. Figure 6B). We therefore concluded a stimulatory effect of EV-associated ROR1 and ROR2 on cancer cell invasion requiring the presence of RhoA without significantly upregulating its expression.We next analyzed whether EV uptake was required for the invasion-promoting effect of ROR-EVs. EV uptake can occur via different pathways and generally the dynamin-2 inhibitor dynasore was shown to achieve the most effective blockade of this process [29, 30]. To confirm that dynasore can efficiently block EV uptake, MCF-7 were pretreated with dynasore for 2 h and stimulated for 24 h with PKH26-labeled lEVs and sEVs from MCF-7 cells. As demonstrated by confocal microscopy, dynasore treatment strongly reduced the uptake of both PKH26-labeled EV populations (Fig. 4D). To confirm this finding, MCF-7-derived EVs were stained with the lipophilic dye DiR that only emits near-infrared fluorescence upon integration into lipid membranes. DiR staining had no influence on EV size or marker expression as determined by NTA and western blot characterization (Suppl. Figure 7). The dye-only control that was prepared according to the same staining protocol, but without the addition of EVs, did not show any measurable particles or protein expression in the analyses (Suppl. Figure 7). Pre-treatment of MCF-7 cells for 2 h with dynasore prior to stimulation with DiR-stained EVs for 24 h significantly decreased the cellular DiR fluorescence, and thus lEV and sEV uptake, as measured by flow cytometry (Suppl. Figure 8). The dye-only control generated only barely detectable background fluorescence. When pre-treating breast cancer cells with dynasore in Boyden chamber assays, however, this had no effect on the pro-invasive properties of the ROR-EVs (Fig. 4E). Thus, our analyses demonstrate that tumor EVs carrying either ROR1 or ROR2 support an aggressive phenotype in cancer cells even after efficiently inhibiting their uptake into target cells.

### Loss of ROR1 on EVs reduces their in vivo biodistribution to target sites of breast cancer metastasis

**Fig. 5:**
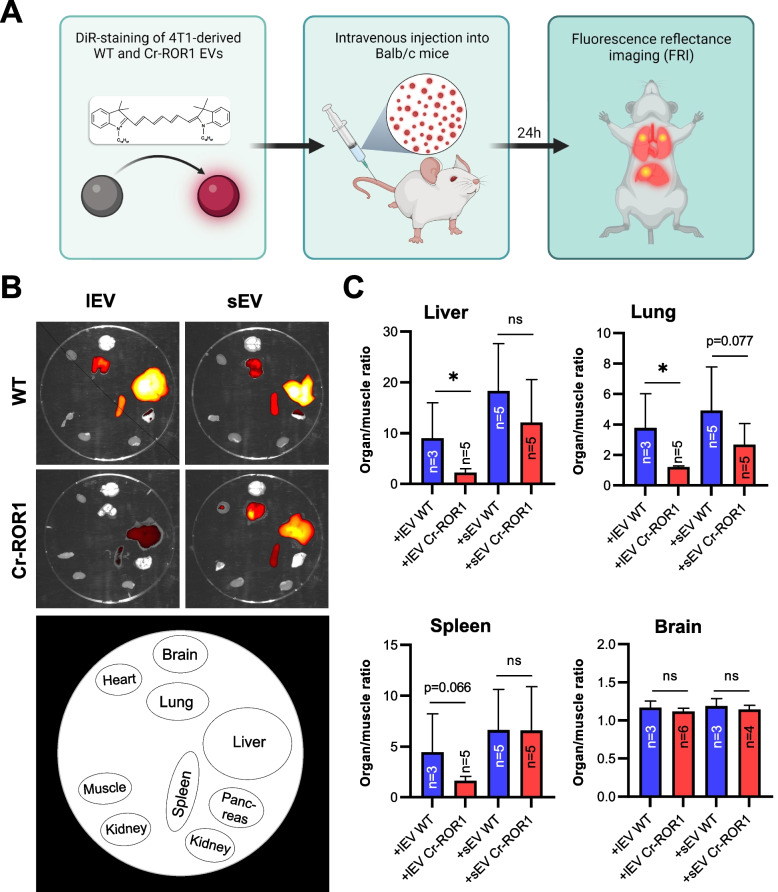
Knockout of ROR1 alters the biodistribution of tumor EVs in mice. **A** Schematic representation of the biodistribution experiments. The figure was created with BioRender.com **B + C** Exemplary ex vivo FRI images (**B**) of mice organs 24 h after the injection of DiR-labeled 4T1 wildtype (WT) or ROR1 knockout (ROR1-KO) EVs and corresponding quantification of the ratio of mean signal intensities organ to muscle (**C**) for selected organs harboring fluorescent signals (mean ± SD). Significance was tested with a one-sided studen’t t test

Once a tumor has gained access to the bloodstream, high numbers of tumor EVs are shed into the circulation and can condition distant organs for subsequent arrival of metastatic tumor cells [11, 12]. The 4T1 breast cancer cell line used in this study preferentially metastasizes to the lung, liver, brain, and bone upon implantation into the mammary fat pad of mice, and thus mirrors the metastatic pattern of human breast cancer [31]. Since our results had revealed that ROR1/2 can be spread via EVs to surrounding cells, we tested whether the presence of ROR1 affects the biodistribution of tumor EVs in the 4T1 mouse model. Since 4T1 cells do not endogenously express ROR2, the analyses were restricted to ROR1. Both lEVs as well as sEVs were isolated from 4T1 wildtype and ROR1 KO cells, stained with DiR and injected in equal amounts into the tail vein of 8–12 week old female Balb/c mice (Fig. 5A). EV biodistribution was analyzed 24 h post injection as this time point had been identified in previous kinetic studies to allow for optimal signal intensities in all EV target organs [23, 32]. FRI scans revealed that fluorescent signals of the EVs were visible in the liver, the lungs as well as the spleen (Fig. 5B), thus confirming these organs as target sites for 4T1 tumor EVs in Balb/c mice [23]. When comparing signal intensities in organs of mice injected with either 4T1 wildtype or ROR1 KO EVs, we observed that in particular ROR1 KO lEVs failed to efficiently reach the liver, lung, and spleen. However, the effect did not quite reach statistical significance for the latter (Fig. 5C). There was a similar trend for an impaired ability of ROR1 KO sEVs to reach the liver and the lungs (Fig. 5C). No differences were observed for tumor EV targeting to the brain in which only minimal fluorescence was detectable. This fits to previous data showing that in contrast to retro-orbital EV injection, the application of the vesicles via the tail vein of the animals does not lead to EV homing to the brain [23, 33]. Taken together, these observations indicate that ROR1 on EVs is involved in directing tumor EVs to future sites of breast cancer metastasis in vivo.

### Levels of ROR1/2 lEVs are elevated in peripheral blood of breast cancer patients and can serve as diagnostic biomarkers

**Fig. 6:**
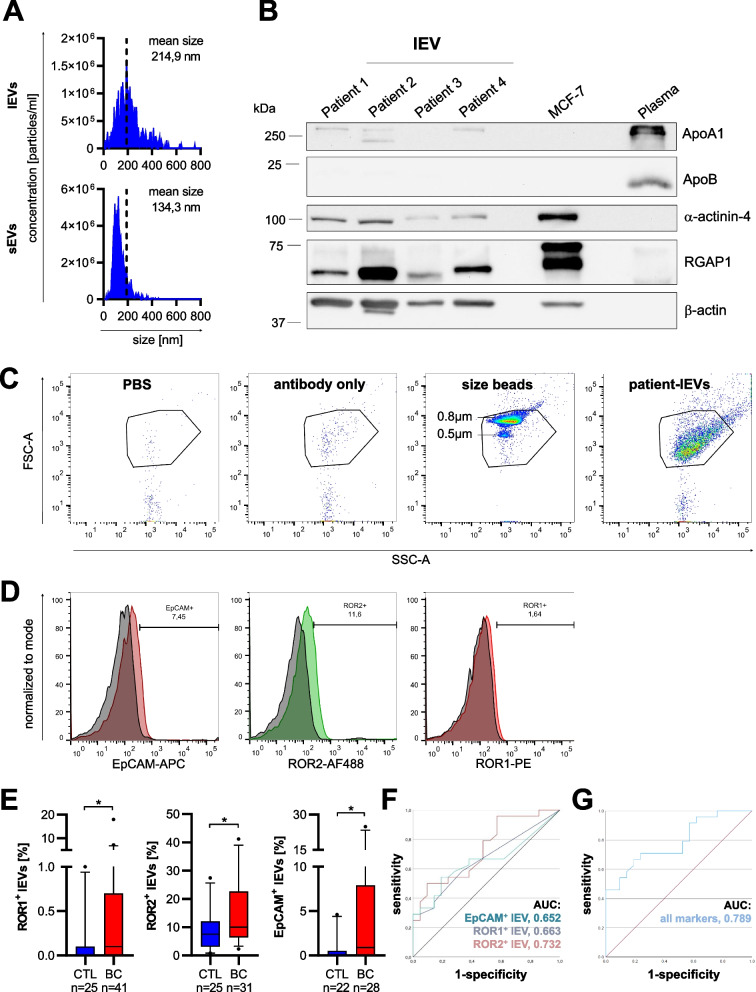
ROR-EVs are novel biomarkers for breast cancer. **A** Representative NTA of lEVs and sEVs isolated from peripheral blood of a breast cancer (BC) patient. **B** lEVs from four BC patients were characterized by western blot for the expression of common lEV markers (α-actinin-4, RGAP1, ꞵ-Actin) or lipoprotein contaminants (ApoA1, ApoB). **C** Flow cytometry of patient-derived lEVs including PBS + 1%EV-depleted FCS, ROR2 antibody-only or size beads as controls. **D + E** The percentage of lEVs carrying the tumor-related antigens ROR1, ROR2, or EpCAM isolated from healthy controls (CTL) or BC patients was measured by flow cytometry. Shown are representatives histograms from one breast cancer patient for each marker (**D**) as well as the quantification from all analyzed samples (**E**). Boxes mark the 25–75 percentiles (line at median) and whiskers the 5–95 percentile. Significance was calculated with a Mann–Whitney test. **F + G** ROC analyses of EV-associated ROR1, ROR2 and EpCAM alone (***E***) or of all three markers combined (**F**)

To confirm that EV-incorporated RORs are also present in peripheral blood of human breast cancer patients, we used our established protocol to isolate lEVs from the plasma of breast cancer patients (*n* = 41) as well as healthy individuals (*n* = 27) (Menck et al., 2017). Patient and control characteristics are listed in Tables 1 and 2. For our analysis, we hypothesized that a certain tumor size and spreading is required for secreting a sufficiently high number of tumor EVs into circulation for subsequent detection. Therefore, we specifically focused on patients with locally advanced and/or metastatic disease in our patient cohort. Since lEVs are larger than sEVs, they can be isolated more rapidly from liquid biopsies and easily be analyzed by standard flow cytometry, a technique well established in clinical routine diagnostics. Hence, quantitative analyses of vesicular protein expression in patient-derived plasma samples were restricted to lEVs. NTA characterization of EVs isolated from the plasma of breast cancer patients showed that the isolated lEVs were substantially bigger than the sEVs isolated from the same serum sample (Fig. 6A). Western blot analysis confirmed the expression of the two lEV marker proteins ɑ-actinin-4 and RGAP1 on patient-derived lEVs, while ApoA1 and ApoB, two common lipoprotein contaminants, were present only at significantly lower amounts compared to pure plasma samples (Fig. 6B). For flow cytometric analysis of patient lEVs a buffer-only control (PBS + 1% EV-depleted FCS) as well as a buffer with antibody control (PBS + 1% EV-depleted FCS + ROR2 antibody) were measured using the same instrument settings as for lEVs and showed only minimal background signals (Fig. 6C). Moreover, small calibration beads with a diameter of 500 and 800 nm were used to define the gate for lEVs and confirmed that most patient-derived lEVs detected by flow cytometry had a size of < 800 nm (Fig. 6C). To exclude swarm detection, we prepared a twofold dilution series in 400 µl PBS with lEVs isolated from three breast cancer patients and recorded the number of events in the lEV gate during 30 s of measurement. Indeed, we were able to reproduce a dilution factor of around 0.5 for all tested dilutions, thus excluding that our flow cytometric measurements of lEVs are influenced by swarm detection (Suppl. Figure 9). Using the thus established protocol for characterizing antigen expression on lEVs by flow cytometry, we measured the percentage of serum lEVs carrying either ROR1, ROR2, or EpCAM. The latter was chosen as a known lEV-associated tumor antigen in breast cancer [22]. Breast cancer patients displayed a significant enrichment of ROR1- (cancer: median 0.1, 95% CI [0.1–1.42]; healthy: median 0, 95% CI [0.004–0.19]), ROR2- (cancer: median 11.55, 95% CI [9.89–18.61]; healthy: median 5.7, 95% CI [4.36–9.35]) as well as EpCAM-positive lEVs (cancer: median 0.9, 95% CI [1.45–7.30]; healthy: median 0.1, 95% CI [0.12–1.28]) compared to healthy controls (Fig. 6D + E). Of note, ROR1- and EpCAM-positive lEVs were barely detectable in healthy controls, suggesting their tumor specificity. Moreover, the expression of both proteins was significantly correlated with each other (*p* = 0.039) (Suppl. Table 1), suggesting that they share a common cell population of origin. In contrast, there was a significant amount of lEVs carrying ROR2 in the control group, indicating its expression on benign lEVs as well. The origin of these ROR2-positive lEVs remains elusive as their levels did not correlate with ROR1-positive lEVs (*p* = 0.134) and only showed a trend for correlation with EpCAM-positive lEVs (*p* = 0.078). None of the three investigated tumor antigens correlated significantly with the number of red blood cells, platelets, or leukocytes in the patient cohort (Suppl. Table 2). In order to assess the diagnostic potential of lEV-associated ROR1, ROR2 and EpCAM, we performed ROC analyses (Fig. 6F + G). The three antigens separated breast cancer patients from healthy individuals with an AUC of 0.663 (95% CI: 0.504–0.821) for ROR1, 0.732 (95% CI: 0.586–0.878) for ROR2 and 0.652 (95% CI 0.489–0.814) for EpCAM. The combination of all three markers discriminated between the two groups with an improved AUC value of 0.789 (95% CI: 0.658–0.919) and confirmed that the definition of tumor EV signatures comprising several markers can enhance the discriminative power of lEV biomarkers as observed in previous studies [22, 34].

## Discussion

EVs are important mediators of intercellular communication that can spread biomolecules over long distances. In this study we detected the Wnt co-receptors ROR1 and ROR2 on EVs from breast cancer cell lines and patients and thus identified a novel mode for spreading Wnt/ROR signaling across tissues. EV-associated ROR1 and ROR2 were involved in directing tumor EVs to potential sites of metastasis in vivo and induced tumor-promoting signaling in recipient tumor cells leading to increased tumor invasion in vitro, which seemed to be dependend on Rho/Rock signaling and potentially independent of EV uptake*.*

This is the first study to describe the presence of ROR1 and ROR2 on lEVs and sEVs. Considering that previous studies have identified the Wnt co-receptor PTK7 on EVs from colorectal cancer cells [35] or FZD10 on sEVs from gastrointestinal cancer cells [36], the vesicular export of Wnt (co-)receptors seems to be a general mechanism. This is not surprising as transmembrane receptors are commonly internalized by receptor-mediated endocytosis and thus enter the endosomal system, where the biogenesis of a large subgroup of sEVs (historically termed “exosomes”) takes place. How RORs might end up on lEVs is less clear. ROR1 has been identified as a scaffold of caveolin-1 [37], which is involved in lEV shedding from the plasma membrane [38], and therefore this association could potentially explain the presence of ROR1 on lEVs. However, since the spatiotemporal localization of the RORs to specialized microdomains of the plasma membrane, which are viewed as platforms for lEV shedding [39], has not been resolved yet, the molecular mechanism of ROR export to lEVs remains elusive. Of note, we did not detect a significant amount of ROR1 or ROR2 on LOs secreted by the investigated breast cancer cells. The mechanisms of LO release are poorly understood at present and therefore the absence of RORs on this EV subpopulation could be either due to diverging biogenesis mechanisms compared to lEVs, or the significant contamination of LO preparations with cell debris as observed in the electron microscopy pictures.

The molecular characterization of various cancers has revealed a considerable degree of intratumoral heterogeneity with distinct cancer cell populations that exhibit genomic and phenotypic diversity [40]. Recent studies have revealed that this applies also to ROR1 and ROR2 as both have been observed to be heterogeneously expressed in pancreatic and mammary tumors in vivo [41–43]. Of note, ROR1-high tumor cells were enriched after chemotherapy and displayed a partial EMT-like phenotype with a higher capacity for tumor growth, metastasis formation and therapy resistance compared to ROR1-low tumor cells [41]. These findings suggest that the concept of transferring ROR proteins to originally ROR-negative tumor cell clones might also be of importance in vivo to spread malignant traits throughout tissues. However, a possible contribution of EVs remains to be demonstrated. The export of ROR1/2 to EVs opens new possibilities for spreading Wnt/ROR signaling to surrounding cells. The concept of horizontal protein transfer via EVs is not new [16–18]. Several studies have proposed that tumor EVs can transfer oncogenic transmembrane receptors between cells in order to promote malignancy of surrounding cells [18, 44]. However, recent observations have underlined that EV uptake does not always equate transfer of functional proteins [45]. While endocytosis is regarded as the main route for EV uptake into recipient cells [19], in their study O’Brien et al.demonstrated that recipient cells can retain internalized EVs in endosomal compartments for days and either target them for lysosomal degradation or re-release [45]. Moreover, most studies label EVs with lipophilic dyes to investigate their incorporation into acceptor cells, which could affect the biochemical properties of the vesicles, and thus their uptake. The question whether intracellular cargo delivery is indeed required for a given EV function is rarely studied. Here, we used unlabeled EVs to study EV-mediated delivery of the RORs and observed that a major part of the ROR-EVs remained attached to the cell surface after 24 h without being taken up. In a previous study we had observed that blocking EV uptake by the dynamin-2 inhibitor dynasore can inhibit the pro-invasive function of tumor EVs on MCF-7 invasiveness by around 40% [29]. However, the additive effect of vesicular ROR overexpression on the tumor EV-mediated increase in cancer cell invasion was not affected by dynasore treatment. This indicates that although the horizontal transfer of some still unknown biomolecules is involved in EV-induced tumor invasion, the interaction of the ROR-EVs with the recipient cell surface seems to be sufficient for the induction of tumor cell invasion. Similar observations have been made particularly for immune responses in which peptide-loaded major histocompatibility complexes on EVs were able to activate cognate receptors on the surface of T cells [15, 46].

Since EV-associated ROR1 and ROR2 do not seem to be integrated into the recipient cell membrane, the question remains which receptors or ligands on the acceptor cell surface serve as interaction partners of EV-RORs to activate invasion-promoting signaling in recipient cells. Typically, binding of Wnt ligands to the RORs initiates receptor homo- or heterodimerization or triggers their association with FZD receptors [3]. Of note, MCF-7 wildtype cells express neither any detectable levels of Wnt ligands [4] nor ROR1/2 raising the question whether ROR-EVs can trigger Wnt ligand-independent signaling. Further research is therefore required to identify ROR cognate receptors in recipient cells.

EVs have great potential as biomarkers in liquid biopsies as they function as platforms that allow the simultaneous detection of several tumor-derived biomolecules on the same vesicle. In this study, we specifically focused on lEVs which had shown the same pro-tumoral function than sEVs in our functional assays. Moreover, lEV isolation and analysis is feasible with standard lab equipment in a timeframe as short as 2 h, thus allowing easy translation of the method to routine clinical diagnostics. Using flow cytometry we detected lEVs carrying ROR1 as well as ROR2 in peripheral blood of breast cancer patients. ROR1-lEVs correlated with EpCAM-lEVs, which have already been established as prognostic and diagnostic biomarkers for breast cancer [22]. In healthy individuals, ROR1 is not expressed on normal mature B cells, plasma cells, or peripheral blood mononuclear cells [3]. Although EpCAM- and ROR1-positive lEVs were almost absent in healthy controls, which might point to their tumoral origin, the identification of their true source requires further research. ROR2, in contrast, has been found on normal CD5^+^ B cells [47] and was readily detectable in all investigated plasma samples. Considering that ROR2-lEVs showed the highest increase in tumor patients compared to controls and provided the best separation between both groups in ROC analyses, an association with the tumor disease seems likely. However, the quite high percentage of ROR2-positive lEVs in the plasma of healthy controls (around 10% of all lEVs) suggests that other cell populations contribute to their secretion into blood. Since a comprehensive analysis of ROR2 expression on the different blood cell populations is lacking so far, the origin of these EVs remains elusive as well.

Current clinical trials evaluate targeting of ROR1/2 in breast cancer as a novel therapeutic approach by using CAR-T cells or the monoclonal antibody cirmtuzumab directed against ROR1 [3]. However, the detection of circulating ROR1-positive EVs in patient plasma in this study could have implications on such endeavors as ROR-EVs could capture the therapeutic antibodies and thereby contribute to therapy failure. Therefore, extracellular ROR1-EVs should be carefully included in therapeutic efforts. This is particularly true as our observations hint towards an important contribution of ROR1 in directing tumor vesicles to future organs of breast cancer metastasis. Although EVs have been identified some time ago as mediators of pre-metastatic niche formation [11, 12], knowledge on specific EV cargo molecules responsible for this metastasis-promoting function is still scarce. Integrins have been demonstrated as critical mediators of EV organotropism in breast cancer [48]. In our analyses, the KO of ROR1 did not seem to have a specific effect on metastatic organotropism as both organs known as frequent sites for metastasis in the 4T1 model, the liver and the lung, showed an equal reduction in EV uptake. This fits to previous observations which demonstrated that the knockdown of ROR1 in MDA-MB-231 breast cancer cells reduced their metastatic spreading to the liver and the lung in immunocompromised mice [49]. The question remains how EV-incorporated ROR1 mediates EV homing to these organs. In the aforementioned study ROR1 knockdown cells injected intravenously into the mice showed less invasion into the lungs [49] suggesting that ROR1 might be involved in extravasation and adhesion, functions which could be mirrored by ROR1-carrying EVs. Furthermore, the traditional ROR ligand Wnt5A as well as the Wnt/ROR downstream targets RhoA/Rock were shown to regulate EV biogenesis [50, 51]. This raises the question whether ROR1 is equally implicated in the regulation of EV cargo, and thus tumor EV uptake or adhesion, at pre-metastatic sites. Endothelial cells, macrophages as well as fibroblasts have been identified as main recipient cell populations of 4T1 EVs in lung parenchyma [33]. However, which of these cell types is mainly affected in its interaction with 4T1-EVs upon loss of ROR1 remains elusive at this point.

## Conclusions

Using breast cancer cell lines and patient samples, this study identified small and large EVs as a novel mode of transportation for spreading ROR1 and ROR2 within the local tumor microenvironment as well as the hematological system where they can be used as cancer biomarkers. EV-incorporated RORs were necessary for the efficient homing of tumor EVs from the circulation to future organs of metastasis in mice. They triggered tumor invasion in recipient breast cancer cells during EV uptake inhibition in vitro, underlining that incorporation of EVs is not always required for their functionality.

## Supplementary Information

**Additional file 1.**

## Data Availability

All data supporting the findings of this study are available within the article and the supplemental information, or from the corresponding author upon reasonable request.

## Abbreviations

AUC: Area under the curve
CI: Confidence interval
EVs: Extracellular vesicles
FCS: Fetal calf serum
FRI: Fluorescence reflectance imaging
KO: Knockout
lEVs: Large extracellular vesicles
LOs: Large oncosomes
NTA: Nanoparticle tracking analysis
ROC: Receiver operating characteristic
sEVs: Small extracellular vesicles
TEM: Transmission electron microscopy

## References

[1] Sung H, Ferlay J, Siegel RL, Laversanne M, Soerjomataram I, Jemal A (2021). Global cancer statistics 2020: GLOBOCAN estimates of incidence and mortality worldwide for 36 cancers in 185 countries. CA Cancer J Clin.

[2] Xu X, Zhang M, Xu F, Jiang S (2020). Wnt signaling in breast cancer: biological mechanisms, challenges and opportunities. Mol Cancer.

[3] Menck K, Heinrichs S, Baden C, Bleckmann A (2021). The WNT/ROR pathway in cancer: from signaling to therapeutic intervention. Cells.

[4] Klemm F, Bleckmann A, Siam L, Chuang HN, Rietkötter E, Behme D (2011). β-catenin-independent WNT signaling in basal-like breast cancer and brain metastasis. Carcinogenesis.

[5] Zhang S, Chen L, Cui B, Chuang H-Y, Yu J, Wang-Rodriguez J (2012). ROR1 is expressed in human breast cancer and associated with enhanced tumor-cell growth. PLoS ONE.

[6] Menck K, Heinrichs S, Wlochowitz D, Sitte M, Noeding H, Janshoff A (2021). WNT11/ROR2 signaling is associated with tumor invasion and poor survival in breast cancer. J Exp Clin Cancer Res.

[7] Gross JC, Chaudhary V, Bartscherer K, Boutros M (2012). Active Wnt proteins are secreted on exosomes. Nat Cell Biol Nature Publishing Group.

[8] Menck K, Klemm F, Gross JC, Pukrop T, Wenzel D, Binder C (2013). Induction and transport of Wnt 5a during macrophage-induced malignant invasion is mediated by two types of extracellular vesicles. Oncotarget.

[9] Yáñez-Mó M, Siljander PR-M, Andreu Z, Zavec AB, Borràs FE, Buzas EI (2015). Biological properties of extracellular vesicles and their physiological functions. J Extracell Vesicles.

[10] Ciardiello C, Cavallini L, Spinelli C, Yang J, Reis-Sobreiro M, de Candia P (2016). Focus on extracellular vesicles: new frontiers of cell-to-cell communication in cancer. Int J Mol Sci.

[11] Peinado H, Alečković M, Lavotshkin S, Matei I, Costa-Silva B, Moreno-Bueno G (2012). Melanoma exosomes educate bone marrow progenitor cells toward a pro-metastatic phenotype through MET. Nat Med.

[12] Costa-Silva B, Aiello NM, Ocean AJ, Singh S, Zhang H, Thakur BK (2015). Pancreatic cancer exosomes initiate pre-metastatic niche formation in the liver. Nat Cell Biol.

[13] Eguchi T, Sheta M, Fujii M, Calderwood SK (2022). Cancer extracellular vesicles, tumoroid models, and tumor microenvironment. Semin Cancer Biol.

[14] Munich S, Sobo-Vujanovic A, Buchser WJ, Beer-Stolz D, Vujanovic NL (2012). Dendritic cell exosomes directly kill tumor cells and activate natural killer cells via TNF superfamily ligands. Oncoimmunology.

[15] Tkach M, Kowal J, Zucchetti AE, Enserink L, Jouve M, Lankar D (2017). Qualitative differences in T-cell activation by dendritic cell-derived extracellular vesicle subtypes. EMBO J.

[16] Rozmyslowicz T, Majka M, Kijowski J, Murphy SL, Conover DO, Poncz M (2003). Platelet- and megakaryocyte-derived microparticles transfer CXCR4 receptor to CXCR4-null cells and make them susceptible to infection by X4-HIV. AIDS.

[17] Ratajczak J, Miekus K, Kucia M, Zhang J, Reca R, Dvorak P (2006). Embryonic stem cell-derived microvesicles reprogram hematopoietic progenitors: evidence for horizontal transfer of mRNA and protein delivery. Leukemia.

[18] Al-Nedawi K, Meehan B, Micallef J, Lhotak V, May L, Guha A (2008). Intercellular transfer of the oncogenic receptor EGFRvIII by microvesicles derived from tumour cells. Nat Cell Biol.

[19] Gurung S, Perocheau D, Touramanidou L, Baruteau J (2021). The exosome journey: from biogenesis to uptake and intracellular signalling. Cell Communication and Signaling.

[20] Ran FA, Hsu PD, Wright J, Agarwala V, Scott DA, Zhang F (2013). Genome engineering using the CRISPR-Cas9 system. Nat Protoc.

[21] Van Deun J, Mestdagh P, Agostinis P, Akay Ö, Anand S, Anckaert J (2017). EV-TRACK: transparent reporting and centralizing knowledge in extracellular vesicle research. Nat Methods.

[22] Menck K, Bleckmann A, Wachter A, Hennies B, Ries L, Schulz M (2017). Characterisation of tumour-derived microvesicles in cancer patients’ blood and correlation with clinical outcome. J Extracell Vesicles.

[23] Gerwing M, Kocman V, Stölting M, Helfen A, Masthoff M, Roth J (2020). Tracking of tumor cell-derived extracellular vesicles in vivo reveals a specific distribution pattern with consecutive biological effects on target sites of metastasis. Mol Imaging Biol.

[24] Pukrop T, Klemm F, Hagemann T, Gradl D, Schulz M, Siemes S (2006). Wnt 5a signaling is critical for macrophage-induced invasion of breast cancer cell lines. Proc Natl Acad Sci U S A.

[25] O’Connell MP, Marchbank K, Webster MR, Valiga AA, Kaur A, Vultur A (2013). Hypoxia induces phenotypic plasticity and therapy resistance in melanoma via the tyrosine kinase receptors ROR1 and ROR2. Cancer Discov.

[26] Kowal J, Arras G, Colombo M, Jouve M, Morath JP, Primdal-Bengtson B (2016). Proteomic comparison defines novel markers to characterize heterogeneous populations of extracellular vesicle subtypes. Proc Natl Acad Sci U S A.

[27] Minciacchi VR, You S, Spinelli C, Morley S, Zandian M, Aspuria P-J (2015). Large oncosomes contain distinct protein cargo and represent a separate functional class of tumor-derived extracellular vesicles. Oncotarget.

[28] Bleckmann A, Conradi L-C, Menck K, Schmick NA, Schubert A, Rietkötter E (2016). β-catenin-independent WNT signaling and Ki67 in contrast to the estrogen receptor status are prognostic and associated with poor prognosis in breast cancer liver metastases. Clin Exp Metastasis.

[29] Menck K, Scharf C, Bleckmann A, Dyck L, Rost U, Wenzel D (2015). Tumor-derived microvesicles mediate human breast cancer invasion through differentially glycosylated EMMPRIN. J Mol Cell Biol.

[30] Toribio V, Morales S, López-Martín S, Cardeñes B, Cabañas C, Yáñez-Mó M (2019). Development of a quantitative method to measure EV uptake. Sci Rep.

[31] Pulaski BA, Ostrand-Rosenberg S (2001). Mouse 4T1 breast tumor model. Curr Protoc Immunol.

[32] Wiklander OPB, Nordin JZ, O’Loughlin A, Gustafsson Y, Corso G, Mäger I, et al. Extracellular vesicle in vivo biodistribution is determined by cell source, route of administration and targeting. J Extracell Vesicles. 2015;4:10.3402/jev.v4.26316. 10.3402/jev.v4.26316 PMC4405624 25899407

[33] Ghoroghi S, Mary B, Larnicol A, Asokan N, Klein A, Osmani N (2021). Ral GTPases promote breast cancer metastasis by controlling biogenesis and organ targeting of exosomes. Pfeffer SR, Golemis EA, Hobbs A, editors. eLife.

[34] Shao H, Chung J, Balaj L, Charest A, Bigner DD, Carter BS (2012). Protein typing of circulating microvesicles allows real-time monitoring of glioblastoma therapy. Nat Med.

[35] Choi D-S, Choi D-Y, Hong B, Jang S, Kim D-K, Lee J (2012). Quantitative proteomics of extracellular vesicles derived from human primary and metastatic colorectal cancer cells. J Extracell Vesicles.

[36] Scavo MP, Depalo N, Rizzi F, Ingrosso C, Fanizza E, Chieti A (2019). FZD10 carried by exosomes sustains cancer cell proliferation. Cells.

[37] Yamaguchi T, Lu C, Ida L, Yanagisawa K, Usukura J, Cheng J (2016). ROR1 sustains caveolae and survival signalling as a scaffold of cavin-1 and caveolin-1. Nat Commun.

[38] Ni K, Wang C, Carnino JM, Jin Y (2020). The evolving role of Caveolin-1: a critical regulator of extracellular vesicles. Med Sci.

[39] Pollet H, Conrard L, Cloos A-S, Tyteca D (2018). Plasma membrane lipid domains as platforms for vesicle biogenesis and shedding?. Biomolecules.

[40] Marusyk A, Janiszewska M, Polyak K (2020). Intratumor heterogeneity: the rosetta stone of therapy resistance. Cancer Cell.

[41] Yamazaki M, Hino S, Usuki S, Miyazaki Y, Oda T, Nakao M (2023). YAP/BRD4-controlled ROR1 promotes tumor-initiating cells and hyperproliferation in pancreatic cancer. EMBO J.

[42] Zhang S, Zhang H, Ghia EM, Huang J, Wu L, Zhang J (2019). Inhibition of chemotherapy resistant breast cancer stem cells by a ROR1 specific antibody. Proc Natl Acad Sci U S A.

[43] Roarty K, Pfefferle AD, Creighton CJ, Perou CM, Rosen JM (2017). Ror2-mediated alternative Wnt signaling regulates cell fate and adhesion during mammary tumor progression. Oncogene.

[44] Xie F, Zhou X, Su P, Li H, Tu Y, Du J (2022). Breast cancer cell-derived extracellular vesicles promote CD8+ T cell exhaustion via TGF-β type II receptor signaling. Nat Commun.

[45] O’Brien K, Ughetto S, Mahjoum S, Nair AV, Breakefield XO (2022). Uptake, functionality, and re-release of extracellular vesicle-encapsulated cargo. Cell Rep.

[46] Raposo G, Nijman HW, Stoorvogel W, Liejendekker R, Harding CV, Melief CJ (1996). B lymphocytes secrete antigen-presenting vesicles. J Exp Med.

[47] Yu J, Chen L, Cui B, Widhopf GF, Shen Z, Wu R (2016). Wnt5a induces ROR1/ROR2 heterooligomerization to enhance leukemia chemotaxis and proliferation. J Clin Invest.

[48] Hoshino A, Costa-Silva B, Shen T-L, Rodrigues G, Hashimoto A, Mark MT (2015). Tumour exosome integrins determine organotropic metastasis. Nature.

[49] Cui B, Zhang S, Chen L, Yu J, Widhopf GF, Fecteau J-F (2013). Targeting ROR1 inhibits epithelial-mesenchymal transition and metastasis. Cancer Res.

[50] Li B, Antonyak MA, Zhang J, Cerione RA (2012). RhoA triggers a specific signaling pathway that generates transforming microvesicles in cancer cells. Oncogene.

[51] Ekström EJ, Bergenfelz C, von Bülow V, Serifler F, Carlemalm E, Jönsson G (2014). WNT5A induces release of exosomes containing pro-angiogenic and immunosuppressive factors from malignant melanoma cells. Mol Cancer.

