# Supplementary material for: Extracellular vesicle-associated tyrosine kinase-like orphan receptors ROR1 and ROR2 promote breast cancer progression
Source: Cell Commun Signal. 2023 Jul 10;21:171. doi: 10.1186/s12964-023-01186-1 (PMC10331971; doi:10.1186/s12964-023-01186-1)
Supplement: Supplementary file 2 — Additional file 1. [file 12964_2023_1186_MOESM1_ESM.pdf]

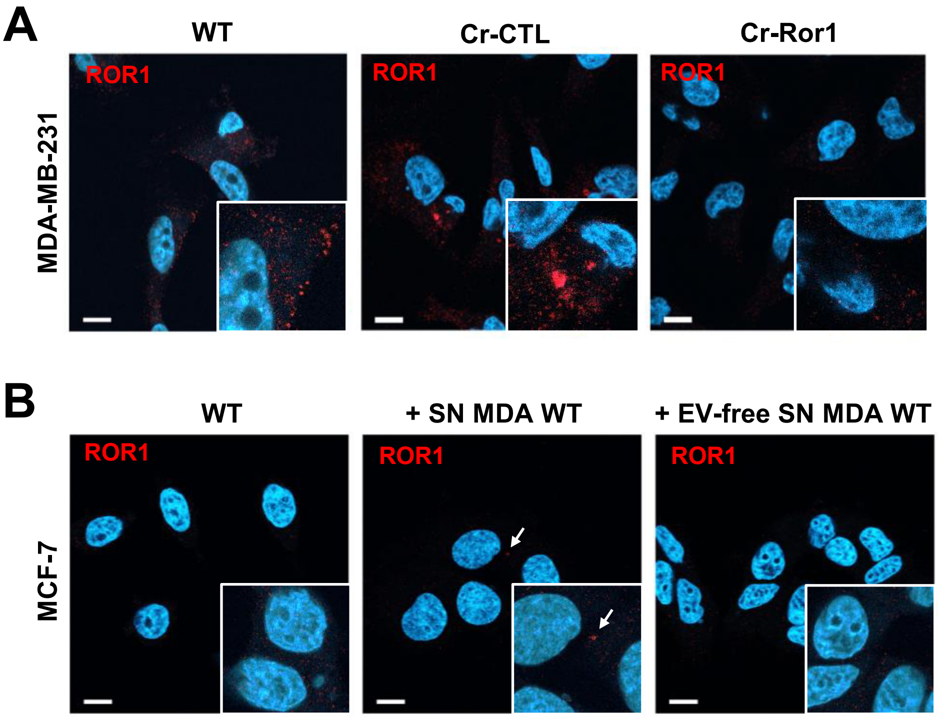

Suppl. Fig. 1: Endogenous ROR1 is delivered by MDA-MB-231-derived EVs to Ror1-negative MCF-7 cells. **A,** Immunofluorescence: ROR1 expression in ROR1-depleted MDA-MB-231 cells (Cr-Ror1) compared to wildtype (WT) and control cells (Cr-CTL). **B,** Immunofluorescence: ROR-negative MCF-7 wildtype (WT) cells were stimulated with supernatant of MDA-MB-231 WT cells (SN MDA WT). For EV-depletion, the SN was centrifuged for 2 h at 143,600 x g. Scale bar: 10 µm.

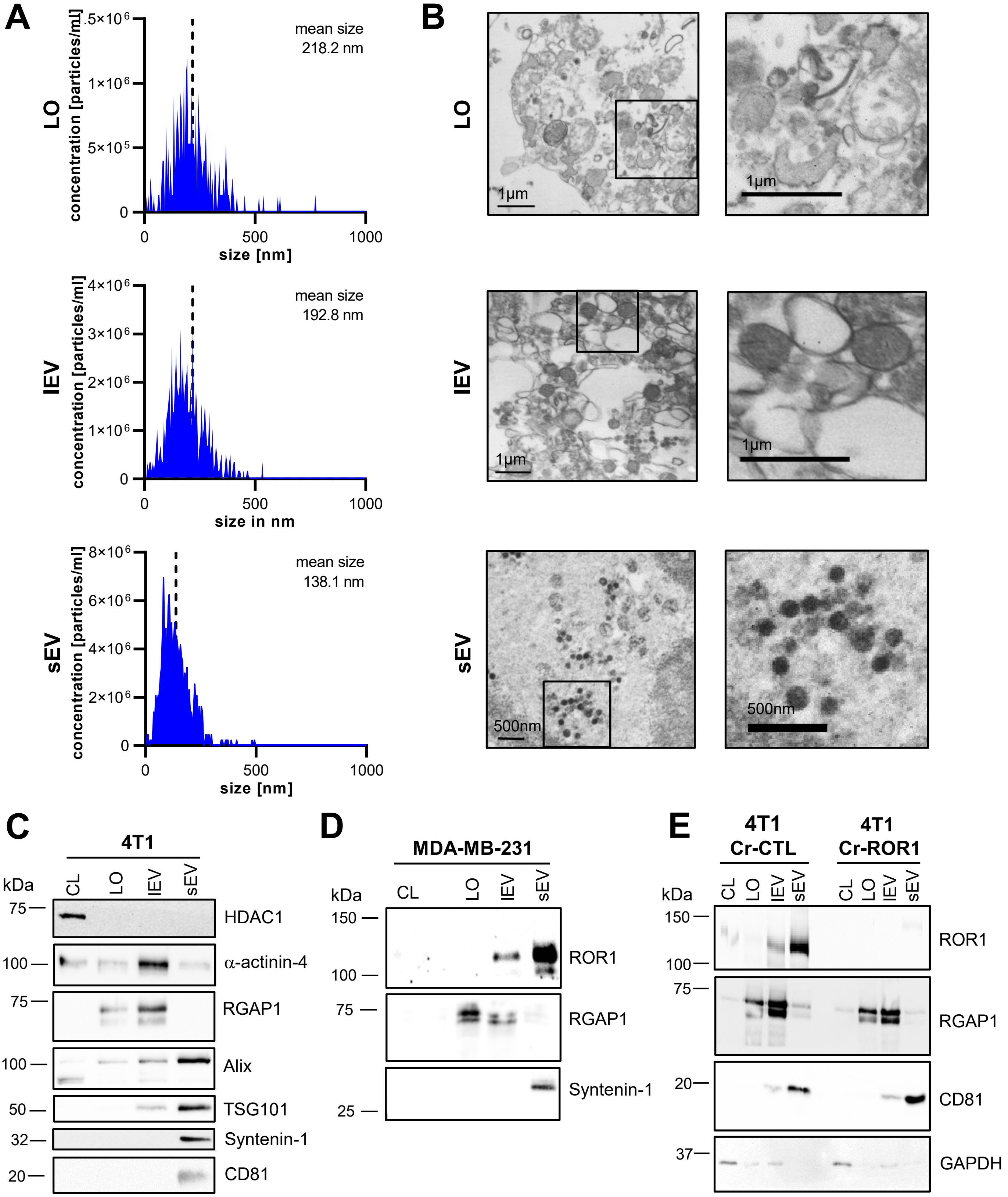

Suppl. Fig. 2: ROR1 is enriched on lEVs and sEVs from 4T1 and MDA-MB-231 cells. ***A,*** NTA of 4T1 EVs. ***B,*** Transmission electron microscopy of 4T1 EVs. The image on the left displays a wide-field overview of the sample. The panel on the right contains a close-up of the area marked with a black box in the wide-field image. ***C,*** Western blot: Characterization of 4T1 EVs for common markers for the different EV subpopulations. GM130 served as a negative marker. Equal amounts of protein were loaded in every lane. ***D,*** Western blot: Characterization of MDA-MB-231 EVs for ROR1 expression on the different EV subpopulations. RGAP1 is included as LO/lEV and Syntenin-1 as sEV marker. Equal amounts of protein were loaded in every lane. ***E,*** Expression of ROR1 on the different EV subpopulations of 4T1 cells transfected with CRISPR/Cas9 control vectors (Cr-CTL) or CRISPR/Cas9 vectors targeting ROR1 (Cr-ROR1) was determined by western blot. RGAP1 served as a marker for LOs and lEVs, CD81 as a marker for sEVs. Equal amounts of protein were loaded in every lane.

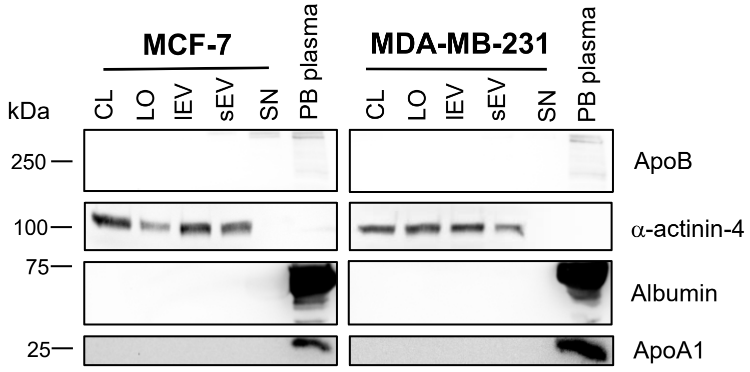

Suppl. Fig. 3: Absence of lipoproteins in isolated EV populations from MCF-7 and MDA-MB-231 cells. Western blot: Lipoprotein expression shown for ApoA1, ApoB and Albumin in different EV populations from MCF-7 and MDA-MB-231 cells compared to respective cell lysates (CL). Cell culture supernatant after sEV depletion (SN) and patient-derived plasma samples (PB plasma) were included as controls. α-actinin-4 served as EV-marker. Equal amounts of protein (10µg) were loaded in every lane.

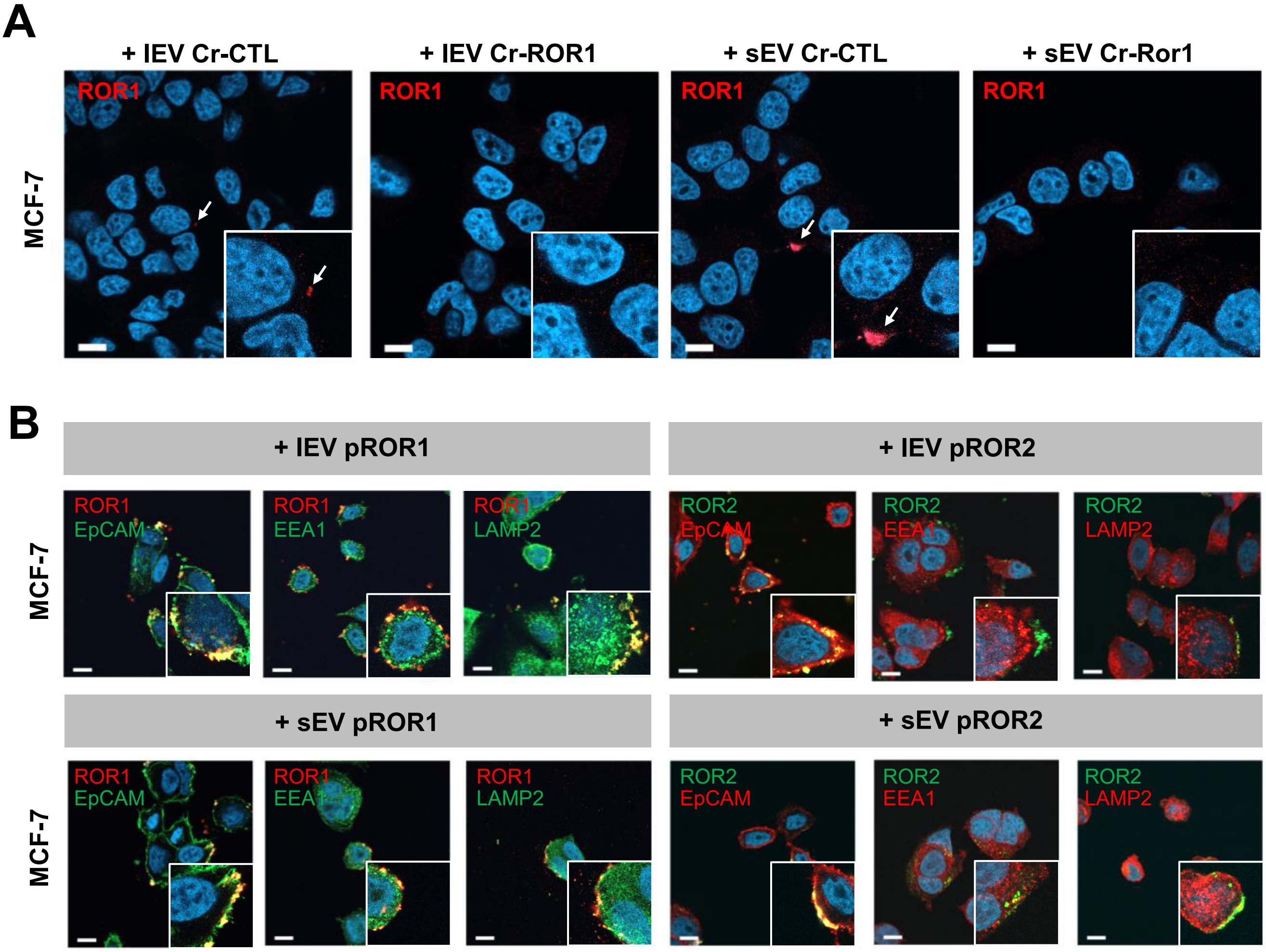

Suppl. Figure 4: ROR1/ROR2 expression after EV-mediated transfer is mainly located at cellular membranes. A, Immunofluorescence: ROR1 expression in MCF-7 cells stimulated for 24h with 10 µg/ml lEVs or sEVs from ROR1-depleted MDA-MB-231 cells (Cr-Ror1) and control cells (Cr-CTL). Arrows indicate EV-delivered ROR1 expression. Scale bar: 10 µm. **B,** Immunofluorescence: Co-localization of ROR1 and ROR2 with EpCAM, EEA1 or LAMP2 in MCF-7 cells stimulated for 4h with 10 µg/ml lEVs (upper panel) or sEVs (lower panel) isolated from MCF-7 pROR1 or pROR2 cells. Scale bar: 10 µm.

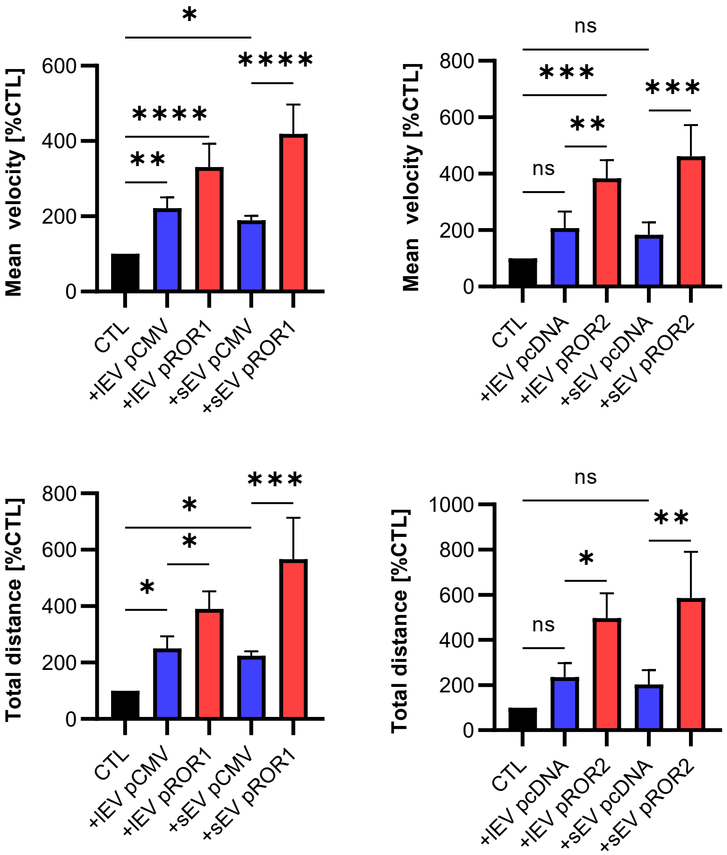

Suppl. Figure 5: EV-associated ROR1 and ROR2 enhance breast cancer cell migration. MCF-7 cells were seeded on ECM and stimulated for 8 h with 10 µg/ml EVs isolated from MCF-7 pROR1, pROR2 or the respective empty vector control cells (mean±SD, n=3). Single cell migration was assessed by live cell microscopy and was analyzed with ImageJ.

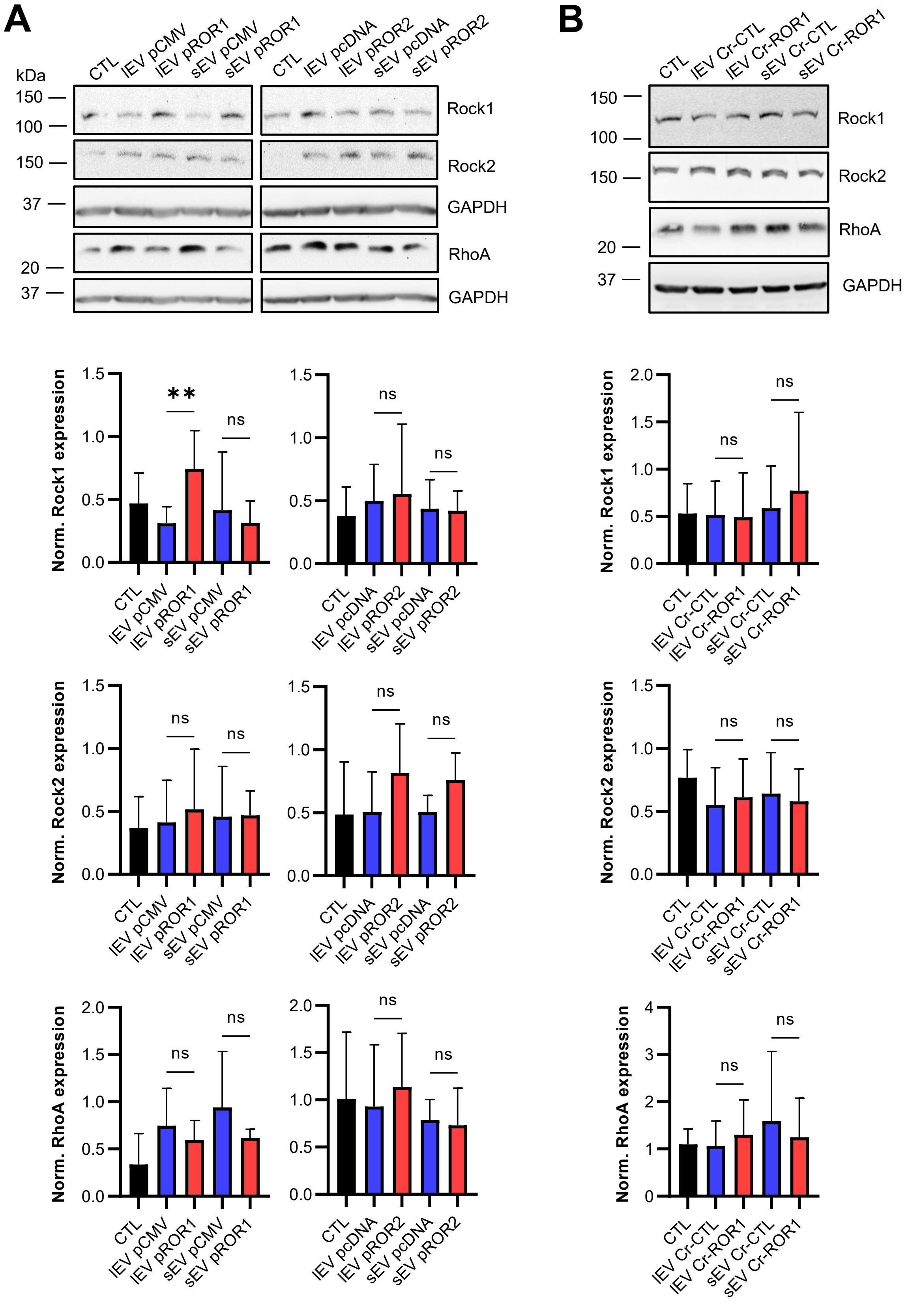

Suppl. Figure 6: EV-delivered ROR proteins have minor effects on the expression of proteins associated to RhoA signaling in target cells. A+B, Western blot: Expression of Rock1, Rock2 and RhoA in (A) MCF-7 cells stimulated for 24h with 50 µg/ml EVs from empty vector (pCMV/pcDNA) and ROR1/2 overexpressing (pROR1/pROR2) cells or (B) EVs from Ror1-deficient MDA-MB-231 cells (Cr-ROR1) and control cells (Cr-CTL), respectively, (upper panels). Equal amounts of protein were loaded in every lane. Signals were quantified by densitometry and normalized to GAPDH expression (lower panels) (mean±SD, n=5).

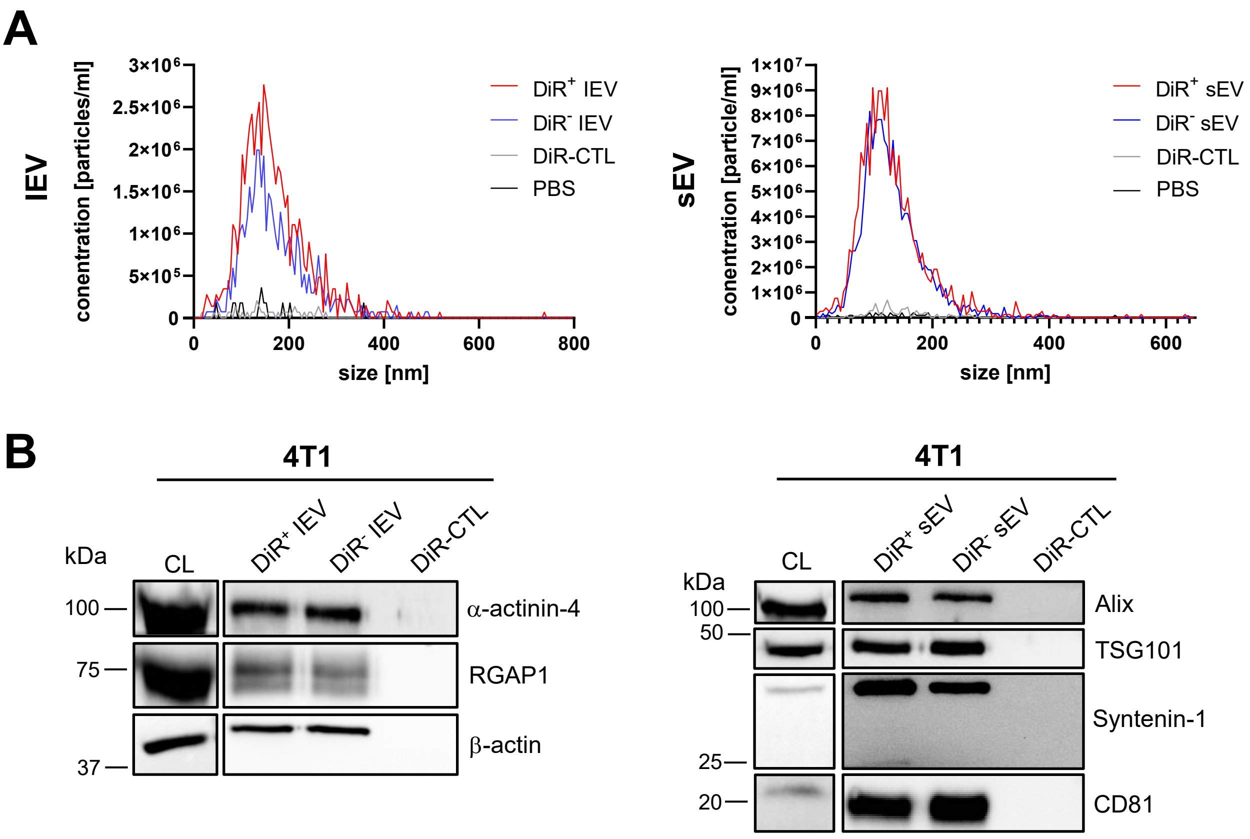

Suppl. Figure 7: DiR staining has no effect on EV size or marker expression. *A+B*, 4T1 lEVs and sEVs were stained with the lipophilic dye DiR and analyzed by (A) NTA or (B) western blot for the expression of common EV markers compared to cell lysates (CL) and dye-only controls (DiR-CTL). For the cell lysates 50 µg and for EV samples 5 µg per lane were loaded.

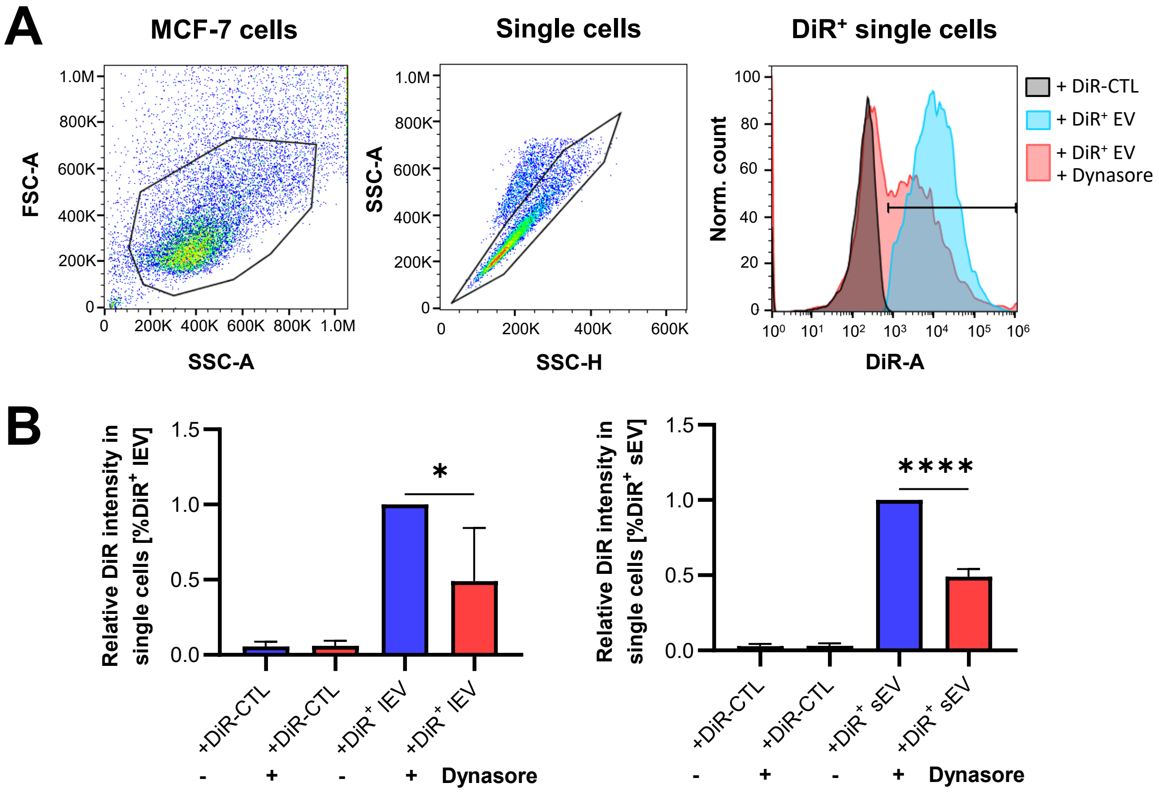

Suppl. Figure 8: Dynasore inhibits EV uptake in MCF-7 cells. ***A,*** Gating strategy of flow cytometric analysis of MCF-7 cells pre-treated with/without Dynasore (12.5 µM) for 2 h and stimulated with DiR-labeled EVs from MCF-7 cells or dye-only controls (DiR-CTL) for 24h. ***B,*** Quantification of the mean DiR intensity in single cells stimulated with DiR-labelled MCF-7 EVs or dye-only control in the presence or absence of Dynasore (mean±SD, n=3).

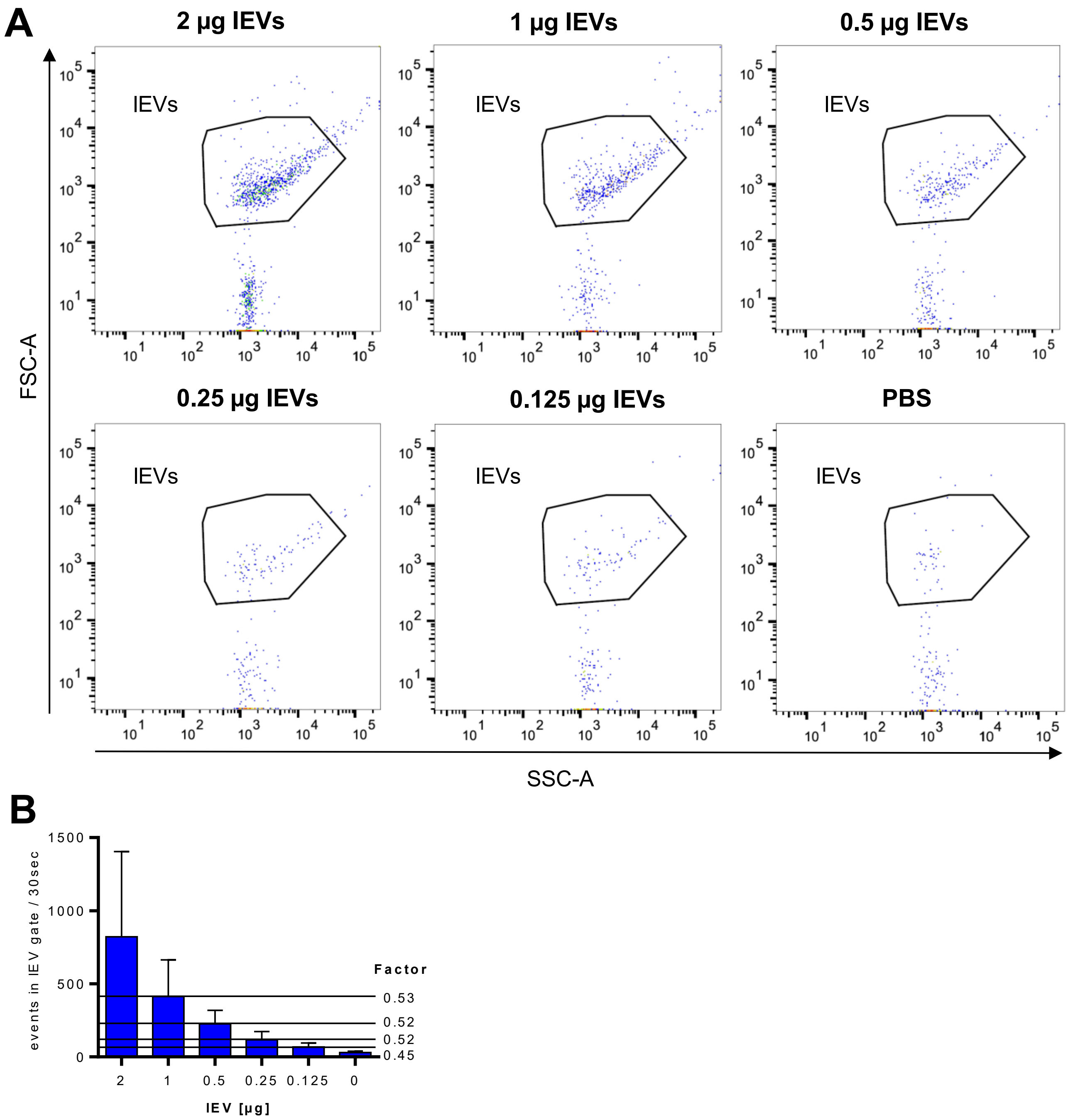

Suppl. Figure 9: IEV signals detected by flow cytometry are not caused by swarm detection. ***A+B,*** Known amounts of IEVs isolated from n=3 different breast cancer patients were serially diluted in equal volumes of PBS and the number of events recorded in the IEV gate during 30 sec of measurement was determined by flow cytometry. Shown are representative FSC vs SSC plots from one patient *(A)* and the quantification from all measurements *(B)* (mean±SD). The mean calculated dilution factor is shown on the right.

Suppl. Table 1: Correlation of the lEV-associated tumor markers with each other in breast cancer patients. For each correlation the Pearson coefficient is indicated with its corresponding p-value. Values shown in bold indicate significant correlations (p<0.05).

Tumor marker	EpCAM+ lEVs	ROR1+ lEVs
**ROR1+ lEVs**	0.399<br>**p=0.039**<br>n=27	
**ROR2+ lEVs**	0.359<br>p=0.078<br>n=25	0.280<br>p=0.134<br>n=30

Suppl. Table 2: Correlation of the lEV-associated tumor markers with blood cell counts in breast cancer patients. For each correlation the Pearson coefficient is indicated with its corresponding p-value.

Tumor marker	Leukocytes $[10^6/\mu l]$	Red blood cells $[10^3/\mu l]$	Platelets $[10^6/\mu l]$
**EpCAM$^+$ lEVs**	0.268<br>p=0.185<br>n=26	-0.179<br>p=0.382<br>n=26	-0.100<br>p=0.625<br>n=26
**ROR1$^+$ lEVs**	-0.002<br>p=0.990<br>n=39	-0.081<br>p=0.622<br>n=39	-0.120<br>p=0.468<br>n=39
**ROR2$^+$ lEVs**	0.105<br>p=0.588<br>n=29	0.064<br>p=0.743<br>n=29	0.000<br>p=0.998<br>n=29
